# Sp Transcription Factors Establish the Signaling Environment in the Neuromesodermal Progenitor Niche During Axial Elongation

**DOI:** 10.1101/2025.06.03.657492

**Published:** 2025-06-04

**Authors:** Ravindra B. Chalamalasetty, Haley Tran, Ryan Kelly, Samuel Kuo, Mark W. Kennedy, Moonsup Lee, Sara Thomas, Nikolaos Mandalos, Vishal Koparde, Francisco Pereira Lobo, Terry P. Yamaguchi

**Affiliations:** 1 Cancer and Developmental Biology Laboratory, Center for Cancer Research, NCI-Frederick, NIH, Frederick, MD, 21702, USA.; 2 Thermofisher Scientific, Frederick, MD, USA; 3 CCR Collaborative Bioinformatics Resource, Center for Cancer Research, National Cancer Institute, National Institutes of Health, Bethesda, MD, 20892, USA; 4 Advanced Biomedical Computational Sciences, Frederick National Laboratory for Cancer Research, Leidos Biomedical Research, Inc., Frederick, MD, 21701, USA

**Keywords:** NMC, epiblast, Wnt/β-catenin, gastrulation, differentiation, stem cell, transcription, axial progenitor

## Abstract

Neuromesodermal competent progenitors (NMCs) are located in the caudal epiblast near the node and primitive streak and give rise to spinal cord and somitic mesoderm during trunk and tail elongation. Their self-renewal depends on an autoregulatory loop involving Wnt3a and Fgf signaling, and the Tbxt and Cdx transcription factors, but the mechanisms underlying loop formation and the establishment of the niche are poorly understood. Here, we identify the zinc-finger transcription factors *Sp5* and *Sp8* (*Sp5/8*) as essential regulators of NMC maintenance. *Sp5/8* expression is controlled by Wnt, Fgf and retinoic acid signaling, and they cooperate with Tbxt, Tcf7 and Cdx2 to sustain a robust autoregulatory network that promotes high Wnt/Fgf and low retinoic acid activity in the niche. These factors bind a novel enhancer essential for *Wnt3a* expression and feedback-loop integrity. Mechanistically, Sp5/8 regulate the dynamic exchange of activating and repressive Tcf complexes at Wnt-responsive enhancers. Our findings define a transcriptional module centered on Sp5/8 that stabilizes niche signaling and transcriptional circuitry essential for NMC fate decisions and trunk development.

## Introduction

The formation of the mammalian trunk and tail depends on a population of self-renewing axial progenitors located at the posterior end of the embryo, which continuously supply new cells for body axis elongation (Aires et al., 2018; Schnirman et al., 2023; Steventon and Martinez Arias, 2017; Wilson et al., 2009). Known as neuromesodermal competent progenitors (NMCs) or neuromesodermal progenitors (NMPs; see Binagui-Casas et al., 2021 for discussion), they reside in the caudal lateral epiblast (CLE) near the primitive streak (PS) at the node-streak border (NSB), and contribute to both neural (spinal cord) and mesodermal (somite) lineages (Binagui-Casas et al., 2021; Cambray and Wilson, 2002, 2007; Garriock et al., 2015; Gouti et al., 2014; Henrique et al., 2015; Tzouanacou et al., 2009; Wymeersch et al., 2016). Their fate is determined by the relative expression of the transcription factors (TFs) Tbxt (Brachyury), which promotes mesoderm, and Sox2, which promotes neural identity (Garriock et al., 2015; Koch et al., 2017; Martin and Kimelman, 2012; Tsakiridis et al., 2014; Wymeersch et al., 2016). Co-expression of both factors maintains NMC bipotency (Wymeersch et al., 2016).

The canonical Wnt/β-catenin signaling pathway plays a central role in regulating *Tbxt* levels and, consequently, NMC fate. In the absence of Wnt ligands, β-catenin is degraded by the destruction complex, and repressive Tcf/Lef transcription factors (TFs) such as Tcf7l1(Tcf3) inhibit Wnt target genes through interactions with Groucho/Tle co-repressors (Bou-Rouphael and Durand, 2021; Ramakrishnan and Cadigan, 2017). Wnt ligand binding stabilizes β-catenin, allowing its nuclear accumulation and activation of target genes, including *Tbxt* (Arnold et al., 2000; Galceran et al., 2001; Yamaguchi et al., 1999), via interaction with activating Tcfs such as Tcf7 and Lef1 at Wnt response elements (WREs).

Genetic studies demonstrate that coordinated Wnt, Fgf and Retinoic Acid (RA) signaling regulates *Tbxt* and *Sox2* expression. Loss of function (LOF) mutations in Wnt/β-catenin pathway components including *Wnt3a, Wnt8a, Ctnnb1* (β-catenin), *Tcf7, Lef1,* or the target genes *Cdx1* and 2, reduce *Tbxt* and increase *Sox2* levels, resulting in the loss of NMCs and ectopic neural tissue (Amin et al., 2016; Anand et al., 2023; Boulet and Capecchi, 2012; Cunningham et al., 2015; Dunty et al., 2008; Greco et al., 1996; Naiche et al., 2011; Takada et al., 1994; van de Ven et al., 2011; Wymeersch et al., 2016; Yamaguchi et al., 1999; Yoshikawa et al., 1997; Zhu and Lohnes, 2022). Similar phenotypes arise in *Fgf4/8* mutants (Boulet and Capecchi, 2012; Naiche et al., 2011) and in embryos lacking *Cyp26a1,* a RA-degrading enzyme expressed in NMCs under the control of Tbxt (Guibentif et al., 2021; Martin and Kimelman, 2010). *Cyp26a1* mutants display increased RA and *Sox2,* reduced *Wnt3a* and *Tbxt,* and impaired axial elongation (Abu-Abed et al., 2001; Abu-Abed et al., 2003; Sakai et al., 2001). Thus, a balance of Wnt, Fgf, and RA signals is essential for NMC maintenance and proper fate specification.

Positive feedback loops further stabilize this signaling environment in the NMC niche. Wnt3a activates *Tbxt* via Tcf7 and Lef1, and in turn, Tbxt enhances *Wnt3a* expression, forming an autoregulatory loop critical for progenitor maintenance (Arnold et al., 2000; Galceran et al., 2001; Koch et al., 2017; Martin and Kimelman, 2012; Yamaguchi et al., 1999). Wnt-induced Cdx factors (especially Cdx2) reinforce this network by co-regulating Wnt and Fgf target genes (Amin et al., 2016; Young et al., 2009; Zhu and Lohnes, 2022), however the molecular basis of Tbxt-Cdx2-mediated regulation of *Wnt3a* remains poorly understood.

Adding further complexity to the Wnt/β-catenin transcriptional network, two zinc-finger TFs from the Specificity protein (Sp) family, Sp5 and Sp8, when mutated display axial truncations phenotypically similar to *Wnt3a* or *Tcf7/Lef1* mutants (Dunty et al., 2014; Galceran et al., 1999; Kennedy et al., 2016) suggesting functional interactions with canonical Wnt effectors. Both factors physically interact with Tcf7 and Lef1 (Kennedy et al., 2016), though their mechanistic roles are unclear. Here, we identify Sp5/8 as key transcriptional regulators in the NMC niche. We show that axial truncation in Sp5/8 mutants results from the loss of trunk NMCs, and that Sp5/8 directly regulate genes in the Wnt, Fgf, and RA pathways. Notably, we uncover a novel enhancer downstream of *Wnt3a* that is co-bound by Sp5/8, Tcf7, Tbxt, and Cdx2, and is required for *Wnt3a* expression. We further show that Sp5/8 stabilize Tcf7 binding at this enhancer, potentially facilitating a switch from repressive Tcf7l1-Tle to activating β-catenin-Tcf7 complexes. Together, our results define a Sp5/8-Tcf7-Tbxt-Cdx2 transcriptional module that sustains high Wnt and Fgf signaling and low RA activity, maintaining axial progenitors and promoting trunk and tail development.

## Results

### *Sp5* and *Sp8* are coexpressed in axial progenitors and are required for trunk development

**Fig.1.**
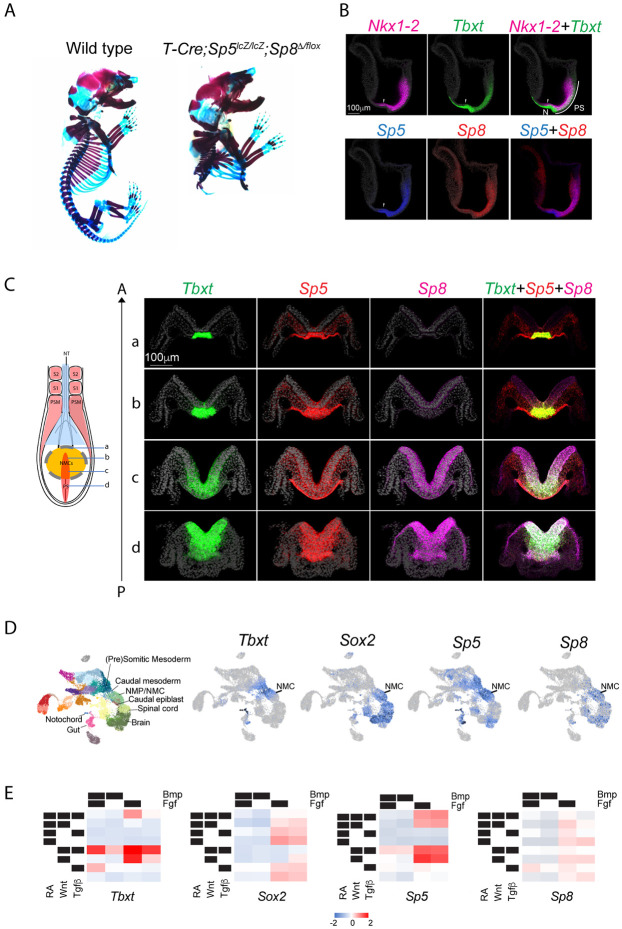
*Sp5* and *Sp8* are coexpressed in axial progenitors and are required for posterior development. A. Alcian blue (cartilage) and Alizarin red (bone) staining of E18.5 skeletons. B. Fluorescent in situ hybridization analysis of *Nkx1-2, Tbxt, Sp5* and *Sp8* expression in E7.75 wildtype embryos. Midline sagittal sections reveal that *Sp5* and *Tbxt* share an anterior boundary in the caudal epiblast (arrowhead). Abbreviations: N, node; PS, primitive streak. C. Whole mount fluorescent in situ hybridization analysis and optical cross sections of E8.5 wild type embryos for *Tbxt* (green), *Sp5* (Red), *Sp8* (purple) and Dapi (grey). The schematic on the left illustrates the axial levels at which optical sections were taken through the node (a), anterior PS/node streak border (b), mid primitive streak (C) and posterior PS (d). D. UMAP plots of single-cell RNAseq analysis of E8.25 mouse embryos showing *Tbxt, Sox2, Sp5*, and *Sp8* transcripts (Pijuan-Sala et al., 2019). E. Heatmaps depicting mean scaled gene expression of *Tbxt, Sox2, Sp5*, and *Sp8* in differentiating ESCs exposed to combinatorial Bmp, Fgf, RA, Wnt and Tgfβ signals (Yeo et al., 2020).

Double null *Sp5*^*lacZ/lacZ*^*;Sp8*^*Δ/Δ*^ mutants die around E14 with severe axial truncations positioned anteriorly compared to either single mutant alone (*Sp8*^*Δ/Δ*^ are tailless, lack limbs, and display neuropore closure defects, *Sp5*^*LacZ/LacZ*^ do not display a phenotype) (Bell et al., 2003; Dunty et al., 2014; Harrison et al., 2000). Double null mutants exhibit exencephaly, ectopic neural tubes and absence of caudal somitic mesoderm suggesting a requirement for *Sp5* and *Sp8* in the brain and in trunk NMCs. To assess a role for *Sp5* and *Sp8* specifically in NMCs, we used the *T-Cre* transgene, which targets axial progenitors in the CLE and the PS but not the node (Garriock et al., 2015; Perantoni et al., 2005), to conditionally delete *Sp8*^*fl/fl*^ in *Sp5*^*LacZ/LacZ*^ embryos. *T-Cre*; *Sp5*^*LacZ/LacZ*^;*Sp8^fl/fl^* mutants (hereafter referred to as *T-Cre; Sp5/8* dko) survived to birth, thereby circumventing the fetal lethality observed in *Sp5/8* double null mutants (Dunty et al., 2014). Analysis of *T-Cre; Sp5/8* dko skeletons revealed that a few abnormal rostral ribs formed, but all skeletal elements caudal to mid-thoracic level T6 were absent (Fig. 1A). The positioning of this axial truncation suggests that *Sp5/8* are redundantly required in trunk NMCs.

Fluorescent in situ hybridization chain reaction (HCR) (Choi et al., 2018) confirmed that *Sp5* and *Sp8* were coexpressed in trunk NMCs. At E7.75, both genes were expressed in the PS and caudal epiblast, overlapping extensively with the NMC markers *Tbxt* and *Nkx1-2* (Fig. 1B). *Sp5’s* anterior expression boundary closely paralleled *Tbxt* in the epiblast near the NSB (arrowhead, Fig. 1B), while *Sp8* expression overlapped with the pre-neural *Nkx1-2*-positive region (Rodrigo Albors et al., 2018) and extended anteriorly into the neural plate (Fig. 1B) (Dunty et al., 2014). Optical cross-sections revealed that *Sp5* and *Sp8* were coexpressed with *Tbxt* in the medial PS and hindgut at E8.5 but extended further laterally into the epiblast beyond the *Tbxt* domain (Fig. 1C). Single cell RNA-seq analysis of E8.25 embryos (Pijuan-Sala et al., 2019) corroborated the HCR findings. *Sp5* expression mirrored *Tbxt* in NMCs, caudal and presomitic mesoderm, notochord and gut, while *Sp8* closely aligned with *Sox2* in NMCs and neural progenitors of the spinal cord and brain (Fig. 1D). *Sp5* and *Sp8* were coexpressed in NMCs, supporting a dual role in trunk NMC maintenance.

To identify upstream regulators of *Sp5/8,* we tested signaling pathways known to influence NMC specification in vitro. *Sp5* and *Tbxt* were robustly activated in differentiating ESCs (Yeo et al., 2020) by combinatorial mesoderm-promoting (Wnt,Fgf,Tgfβ) signals (Koch et al., 2017; Sudheer et al., 2016), but inhibited by RA, a neural inducer, or Bmp which promotes lateral mesoderm fates from NMC-derived mesoderm (Row et al., 2018) (Fig. 1E). In contrast, *Sp8* was activated by neural-promoting (RA, Fgf) and mesoderm-promoting (Wnt, Fgf) signals, but also repressed by Bmp (Fig. 1E). Thus, *Sp5* and *Sp8* expression are distinctly, yet coordinately, regulated by signals that control NMC specification and differentiation.

### Transcriptional profiling suggests roles for Sp5/8 in the regulatory circuitry of NMCs

**Fig.2.**
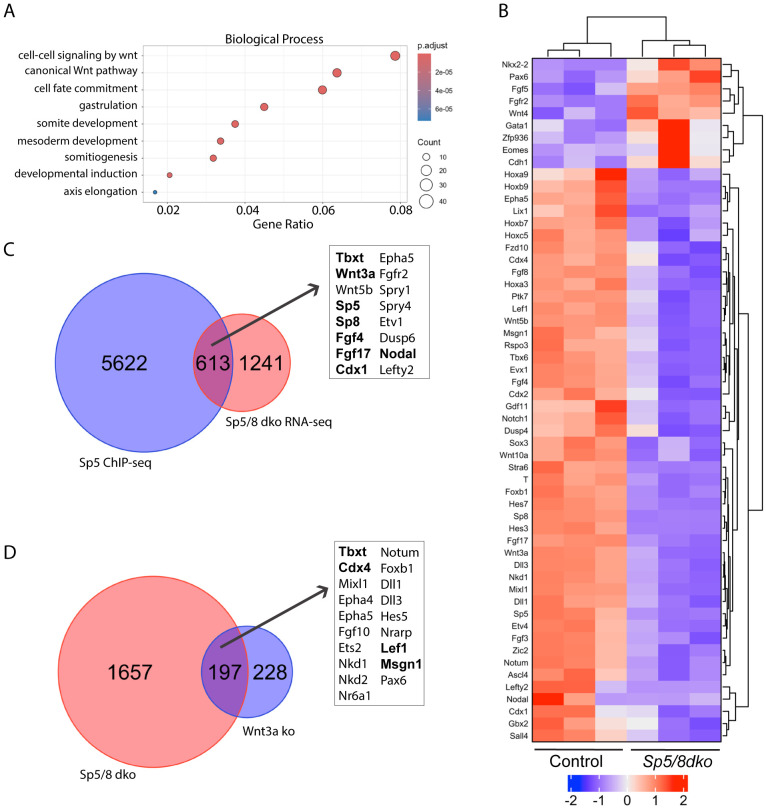
Sp5/8 regulates the Wnt3a/β-catenin pathway. A. Over Representation Analysis (ORA) of 564 downregulated genes in E8.5 *Sp5/8* dko mutants. Color scale reflects adjusted P-value, circle size represents gene number. B. Heat map of log2 z-scores of normalized counts showing differentially expressed genes (P≤0.05; FC ≥0.5). C. Venn diagram illustrating the intersection between Sp5 ChIPseq and *Sp5/8* dko RNA-seq analyses. 613 genes including *Wnt3a* and *Tbxt* are direct targets of Sp5. D. Venn diagram showing 197 differentially expressed genes in common between the *Sp5/8 dko* and *Wnt3a ko* transcriptomes.

**Fig.3.**
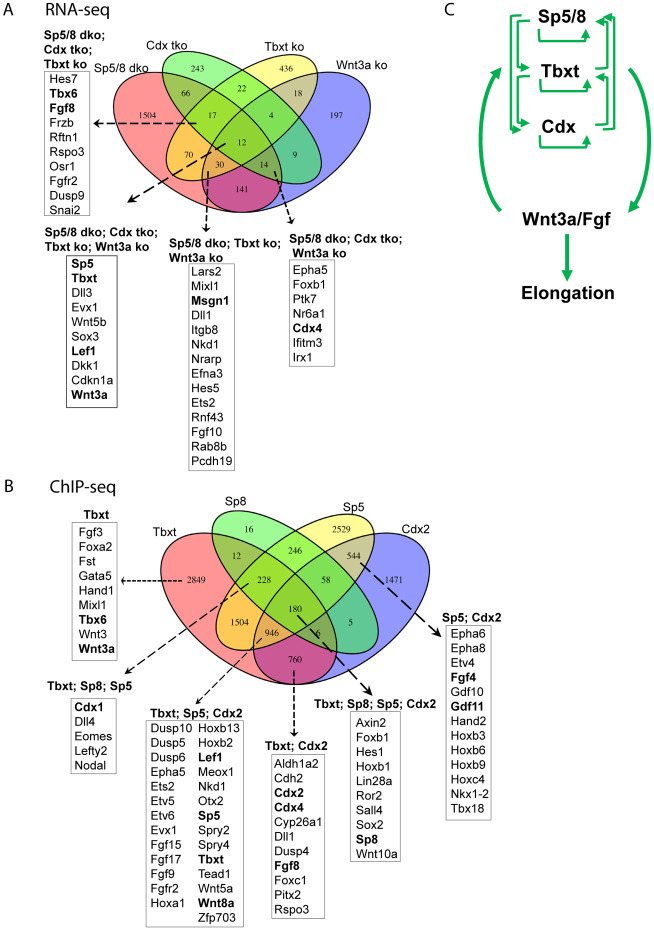
Sp5/8 are integral components of the core regulatory circuitry of axial progenitors. A. Four-way Venn diagram illustrating differentially expressed genes common to *Sp5/8 dko, Cdx tko* (Amin et al., 2016), *Tbxt ko* (Koch et al., 2017), and *Wnt3a ko* transcriptomes. Key regulators are highlighted in bold. B. Four-way Venn diagram illustrating direct target genes bound combinatorially by Tbxt, Flag-Sp5, Flag-Sp8, and/or Cdx2. ChIP-seq was performed in NMC populations differentiated in vitro from CHIR-treated ESCs or, in the case of Cdx2, EpiSCs (Amin et al., 2016). C. Sp5/8, Tbxt and Cdx TFs form self-sustaining autoregulatory loops that collectively maintain a Wnt3a/Fgf positive feedback loop to drive NMC self-renewal and axial elongation.

To identify genes co-regulated by *Sp5* and *Sp8,* bulk RNA-seq was performed on the posterior region of *Sp5/8* double-null embryos at E8.5 (4-6 somite stage (ss)), prior to overt morphological changes (Supp. Fig. 1A, B). Over-Representation Analysis (ORA) of 564 downregulated genes revealed that these genes are associated with canonical Wnt signaling, gastrulation, mesoderm formation, somite development, and axis elongation (Fig. 2A, Supp. Fig. 1C, D, Supp. data file-1). These include TFs known to control NMC specification *(Tbxt, Cdx1, 2, 4*), posterior identity *(Hoxa9, Hoxb9, Hoxb7* and others), and presomitic mesoderm (PSM) differentiation and segmentation *(Lef1, Hes7, Tbx6, and Msgn1*) (Fig. 2B). Additionally, key signaling pathways required for the development of axial progenitors – Wnt/β-catenin *(Wnt3a, Wnt5b, Axin2, Lef1, Cdx1,2,4, Tbxt, Msgn1),* Fgf (*Fgf4, Fgf8, Fgf17, Dusp4, Etv4),* Nodal (*Nodal, Leftyl, Lefty2*) Notch (*Notch1, Dll1, Dll3, Hes7*) and Bmp *(Gdf11*) – were disrupted in mutants (Aires et al., 2019; Dunty et al., 2014; Gouti et al., 2017; Jurberg et al., 2013; Koch et al., 2017; Wymeersch et al., 2019). Many affected genes, notably *Wnt3a, Tbxt, Cdx1* and *Fgf4,* as well as *Sp5* and *Sp8,* were bound by Sp5/8 in ChIP-seq assays performed in differentiating EBs (Fig. 2C, 3B, Supp. data file-1), suggesting that Sp5 and Sp8 autoregulate their own expression, and participate directly in the Wnt3a-Tbxt-Cdx positive feedback loop.

We reasoned that if Sp5 and Sp8 function in the autoregulatory loop then comparative RNA-seq analyses for E8.5 *Sp5/8, Wnt3a, Tbxt* (Koch et al., 2017) and *Cdx1/2/4* (Amin et al., 2016; Zhu and Lohnes, 2022) mutants should reveal common differentially expressed genes (DEGs). A comparison of *Sp5/8* and *Wnt3a* mutant transcriptomes identified a set of 197 genes (68% concordant), including the established Wnt targets *Tbxt, Cdx4, Lef1* and *Msgn1* (Fig. 2D; 3A, Supp. Fig. 1E, Supp. data file-2) (Chalamalasetty et al., 2014; Galceran et al., 2001; Hovanes et al., 2001; Yamaguchi et al., 1999). Notably, *Wnt3a* and *Sp5/8* displayed codependence as *Wnt3a* expression was reduced in *Sp5/8* dko embryos (−2.93 log2FC), while *Sp5* (−1.9 log2FC) and *Sp8* (−0.92 log2FC) levels were reduced in *Wnt3a*^*−/−*^ mutants. A similar codependency was observed between *Sp5/8* and the *Cdx* genes (Supp. Fig. 2A, B), and *Sp5/8* and *Tbxt* (Supp. Fig.2C, D), when the *Sp5/8* dko was compared to *Cdx* tko and *Tbxt* ko transcriptional profiles (Supp. Fig. 2, Supp. data file-3). Further comparisons across all four mutant transcriptomes (*Sp5/8, Wnt3a, Tbxt* and *Cdx1/2/4)* identified a core set of 12 shared DEGs that included *Sp5, Wnt3a, Tbxt,* and *Lef1* (Fig. 3A, Supp. Fig. 2, Supp. data file-3). Examination of Sp5, Sp8, Tbxt and Cdx2 (Amin et al., 2016) ChIP-seq datasets, derived from in vitro-differentiated NMC populations, confirmed that cis-regulatory elements at *Sp5, Sp8, Tbxt, Cdx1, 2,* and *4* were bound directly by Tbxt and either Cdx2, Sp5, or Sp8 (Fig. 3B, Supp. data file-4). The high degree of interaction between these TFs suggests that Sp5 and Sp8 are integral components of the regulatory circuitry of axial progenitors (Fig. 3C).

### NMC and PSM fates depend on Sp5/8 activity in vivo

**Fig.4.**
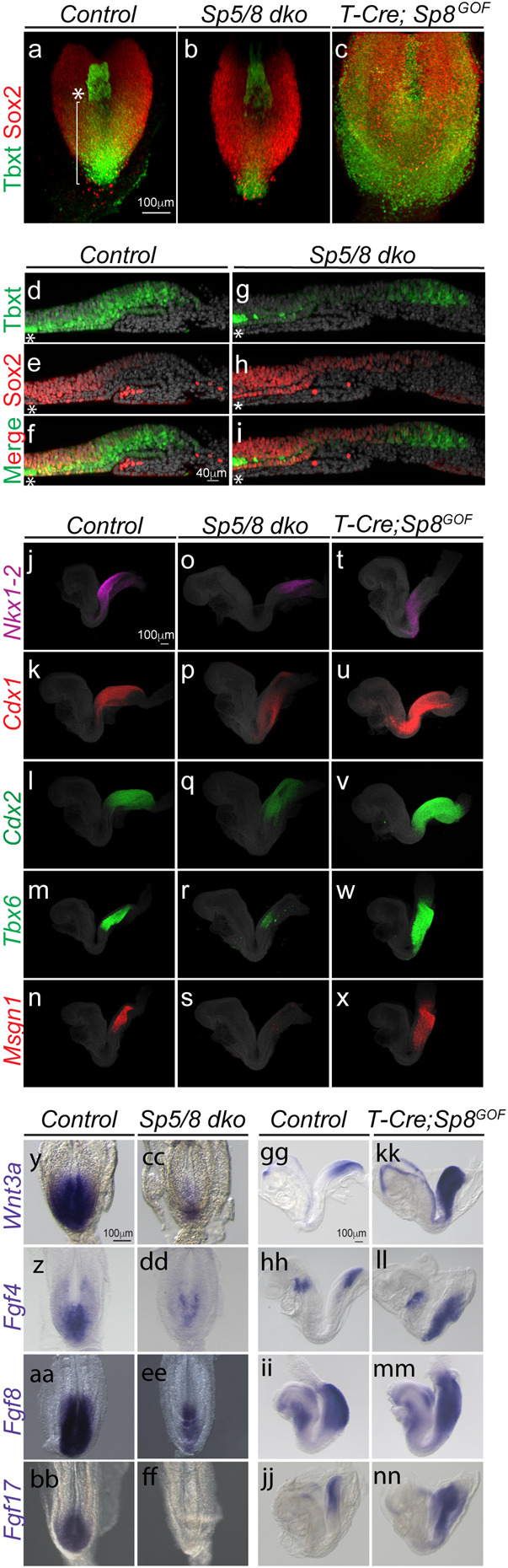
Sp5/8 regulate axial progenitors and the expression of niche factors. A-C. Whole mount immunofluorescence analysis showing Tbxt (green) and Sox2 (red) protein expression in the posterior region of E8.5 control (a), *Sp5/8 dko* (b) and *T-cre; Sp8^gof^* (c) mutants. The bracket indicates the length of the primitive streak, the asterisk depicts the NSB. D-I. Midline sagittal sections taken through the primitive streak of flat mounted E8.5 control (d-f) and *Sp5/8 dko* embryos (g-i) processed for Tbxt (green), and Sox2 (red) IF (Dapi, grey). J-X. HCR analysis of *Nkx1-2* (magenta), *Cdx1* (red), *Cdx2* (green), *Tbx6* (green), and *Msgn1* (red) expression in E8.5 control (j-n), *Sp5/8 dko* (o-s) and *T-cre; Sp8^gof^* (t-x) embryos. Y-NN. Chromogenic WISH of E8.5 control (y-bb) and *Sp5/8 dko* (cc-ff), E8.5 control (gg-jj) and *T-cre; Sp8^gof^* (kk-nn) with DIG-labeled *Wnt3a (y, cc, gg, kk), Fgf4 (z, dd, hh, ll), Fgf8 (aa, ee, ii, m)* and *Fgf17 (bb, ff, jj, nn)* riboprobes. (y-ff) dorsal views, (gg-nn) lateral views.

To characterize the role of Sp5 and Sp8 in the Wnt3a-Tbxt feedback loop and in trunk axial progenitors in vivo, we first examined the expression of the NMC-defining TFs Tbxt and Sox2 in *Sp5/8* dko embryos at the 4-7ss stage when trunk somites are emerging and when axial progenitors are being generated. Whole-mount immunofluorescence (IF) showed that Tbxt was markedly reduced or absent from the anterior PS, while Sox2 was elevated and posteriorly expanded in mutants (Fig. 4b), compared to controls (Fig. 4a). Mid-sagittal sections confirmed that Tbxt+; Sox2+ NMCs were largely found at the NSB (Fig. 4d-f), and that these double positive NMCs were absent in *Sp5/8* dko mutants (Fig. 4g-i). The low Tbxt/high Sox2 levels are consistent with an NMC-to-neural fate conversion in the absence of Sp5/8. In contrast, Sp8 overexpression in axial progenitors dramatically expanded the Tbxt expression domain, promoting overgrowth of the entire posterior region (Fig. 4c; Supp. Fig. 3A). These results show that Tbxt and Sox2 levels are controlled by Sp5 and Sp8.

HCR analysis of NMC (*Nkx1.2, Wnt8a),* posterior identity (*Cdx1, Cdx2*), and PSM markers (*Tbx6, Msgn1*) (Amin et al., 2016; Cunningham et al., 2015; Garriock et al., 2020; Gouti et al., 2017; Rodrigo Albors et al., 2018; Zhu and Lohnes, 2022) revealed their reduction or absence in *Sp5/8* mutants, reinforcing the requirement for Sp5/8 in NMC and PSM maintenance (Fig. 4j-s; Supp. Fig. 3C). Interestingly, overexpression of Sp8 in axial progenitors resulted in reduced expression of the NMC markers *Nkx1.2* and *Wnt8a,* and a dramatic increase in the levels and spatial domains of the Wnt targets *Cdx1, Cdx2, Tbx6* and *Msgn1,* indicating that elevated Sp5/8 activity drives the Wnt pathway and posterior identity, and pushes NMC differentiation towards mesodermal fates (Supp. Fig. 3B, D: Fig. 4t-x). Together, the data suggest that Sp5/8 control NMC fate decisions in a dose-dependent manner; loss of Sp activity disrupts NMC maintenance, promoting neural fate, intermediate levels maintain NMCs, and high Sp5/8 levels favor mesoderm differentiation.

### Sp5/8 directly regulate the signaling environment of the NMC niche

RNA-seq analysis indicated disrupted expression of key genes in the Wnt, Fgf and RA signaling pathways in *Sp5/8* mutants, suggesting that Sp5/8 could control NMC fate decisions by regulating the niche environment (Fig. 2, 3). WISH analysis confirmed that *Wnt8a* (Supp Fig. 3C), *Wnt3a, Fgf4, Fgf8* and *Fgf17,* expressed in the CLE and PS of controls (Fig. 4y-bb) (Boylan et al., 2020; Crossley and Martin, 1995; Cunningham et al., 2015; Yamaguchi and Rossant, 1995) were significantly reduced or absent in *Sp5/8* mutants (Fig. 4cc-4ff). Additionally, expression of *Cyp26a1,* which degrades RA, was also downregulated, while *Aldh1a2* (an RA synthesis enzyme) levels were maintained, implying elevated levels of RA in mutant axial progenitors (Supp. Fig. 4a-f). Collectively, these results show that loss of Sp activity disrupts the balance of morphogens - lowering Wnt and Fgf while increasing RA - thereby impairing NMC self-renewal.

Transient overexpression of Sp8 in axial progenitors led to elevated levels and expanded expression domains of *Wnt3a, Fgf4, Fgf8* and *Fgf17,* consistent with activation of the Wnt3a-Tbxt feedback loop (Fig. 4gg-4nn). Fgf4 and 8 play an important role during axial elongation to maintain the undifferentiated state of presomitic progenitors (Boulet and Capecchi, 2012; Naiche et al., 2011). The increased and expanded expression of *Fgf4* and *8* predicts that the PSM progenitor pool was expanded at the expense of somitic differentiation. Increased expression of the PSM markers *Tbx6* and *Msgn1* (Fig. 4w, x), coupled with the reduced expression domains of the somite markers *Uncx4.1* and *Aldh1a2* (Supp. Fig. 3B, 4c, d), is consistent with a role for Sp8 in regulating the PSM progenitor pool through these Fgfs. These results are consistent with Sp5/8 as direct regulators of niche signals critical for NMC fate decisions.

### An Sp5/8-Tcf7-Cdx-Tbxt TF module regulates *Wnt3a* via a novel downstream enhancer

**Fig.5:**
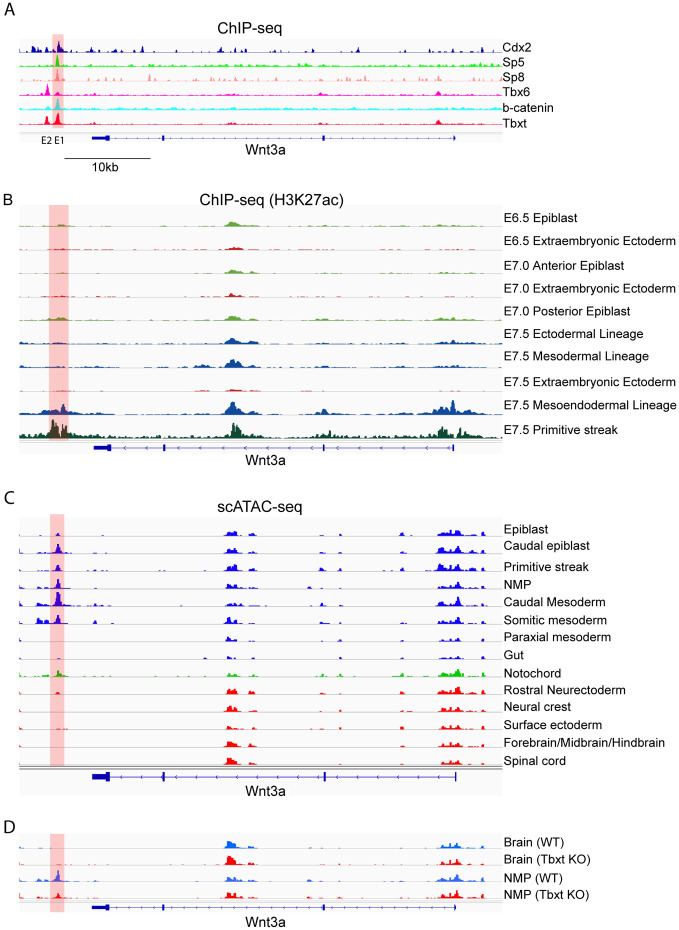
Identification of a novel *Wnt3a* downstream enhancer. A. Integrative Genomics Viewer analysis of ChIP-seq peaks for Cdx2 (Amin et al., 2016), Flag-Sp5, Flag-Sp8, Tbx6, β-catenin, and Tbxt (Koch et al., 2017) at *Wnt3a*. Two putative downstream enhancers are denoted as E1 (red bar, referred to as *Wnt3a^DE^* in text) and E2. B. ChIP-seq tracks derived from (Xiang et al., 2020) and (Yang et al., 2019) showing H3K27ac deposition at both the promoter and putative enhancer region of *Wnt3a* in the mesendodermal and primitive streak lineages. C. scATAC-seq data of E8.5 wild type mouse embryos showing accessibility across *Wnt3a* (Argelaguet et al., 2022). D. scATAC-seq data from wild type (WT) and *Tbxt* knockout (KO) mouse embryos at E8.5 showing reduced accessibility at *Wnt3a^DE^* in the absence of *Tbxt* (Argelaguet et al., 2022).

Given the crucial role that Wnt3a plays in NMC self-renewal, we focused our attention on gaining a mechanistic understanding of how Sp5/8 regulate *Wnt3a*. Examination of the ChIP-seq data for Sp5 and Sp8 binding sites led to the identification of peaks in a conserved, intergenic region 4.2 kb downstream of *Wnt3a* (Fig. 5A; Supp. Fig. 5A). This region bears the hallmarks of an enhancer as it is bound by H3K27ac, an epigenetic mark of active enhancers, in the PS and mesendoderm of E7.5 embryos when *Wnt3a* is first expressed in these tissues, but not in extraembryonic or earlier-staged tissues where it is not expressed (Xiang et al., 2020; Yang et al., 2019) (Fig. 5B). Furthermore, scATAC-seq data from E8.5 mouse embryos showed that while the *Wnt3a* promoter was broadly accessible across all cell types, the putative enhancer element was accessible only in *Wnt3a*-expressing cells (Fig. 5C) (Argelaguet et al., 2022; Pijuan-Sala et al., 2019). This included caudal epiblast, PS, NMP, caudal and somitic mesoderm, and notochord, but not neural, surface ectoderm, and gut which do not express *Wnt3a* at this stage. These findings suggest that this element regulates tissue-specific expression of *Wnt3a*. Hereafter, we refer to this element as the *Wnt3a*^*DE*^ (Downstream Enhancer).

Further mining of ChIP-seq datasets showed that in addition to binding Sp5 and Sp8, the *Wnt3a*^*DE*^ is co-occupied by the NMC regulators β-catenin, Cdx2 and Tbxt but not Tbx6 (Fig. 5A), and contains consensus binding motifs for Sp5, Tcf/Lef, Cdx and Tbx, suggesting direct regulation by these TFs (Supp. Fig. 5B). To examine the functional consequences of Tbxt binding to the *Wnt3a*^*DE*^, we analyzed scATAC-seq data performed on E8.5 *Tbxt*^*−/−*^ embryos (Argelaguet et al., 2022). Loss of *Tbxt* resulted in reduced accessibility of the *Wnt3a*^*DE*^ in NMPs (Fig. 5D) suggesting that Tbxt regulates *Wnt3a* expression in NMCs by controlling chromatin accessibility.

**Fig.6.**
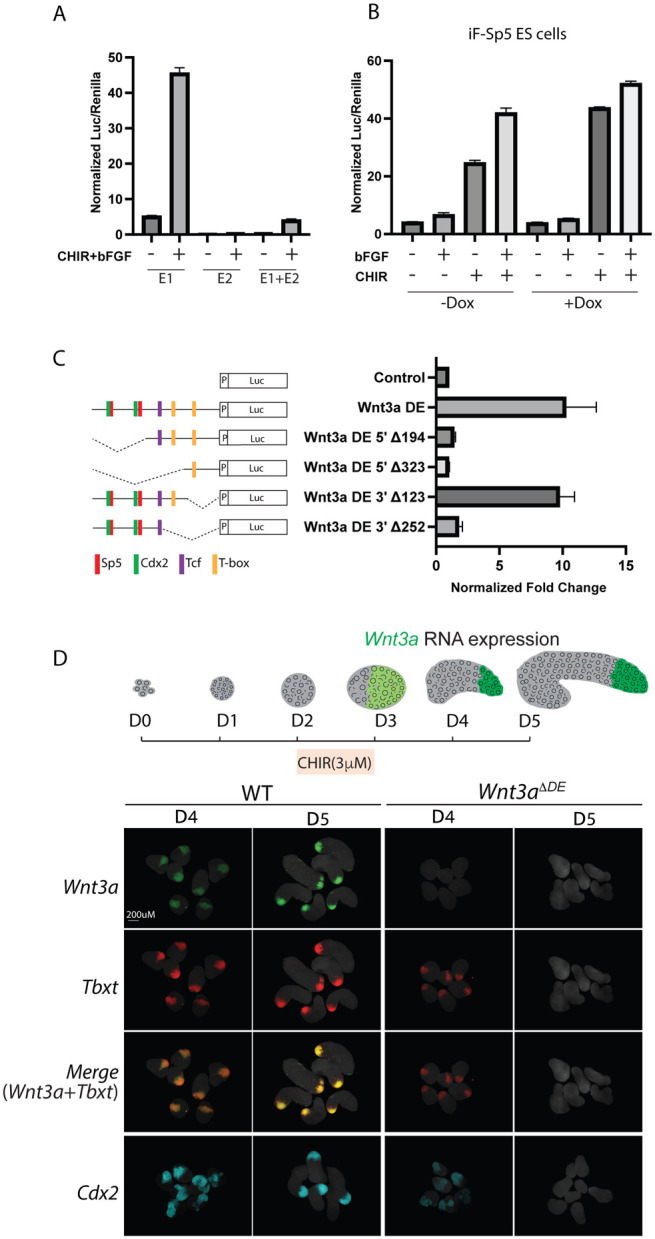
The *Wnt3a^DE^* is a WRE necessary for expression of *Wnt3a* and the *Wnt3a-Tbxt* autoregulatory loop. A. Luciferase reporter assays of *Wnt3a* enhancer constructs: 0.47kb E1 (*Wnt3a^DE^*), 0.8kb E2 and the 1.8kb E1+E2, in differentiating ESCs treated with CHIR and Fgf to generate NMCs. Data are depicted as Mean+/− SD (n=3). Y-axis, relative luciferase units. B. Sp5 overexpression enhances *Wnt3a^DE^*-luc activity in differentiating i3xFlag-Sp5 ES cells treated with bFGF (12ng/ml), CHIR(3μM), or bFGF+CHIR +/− Dox to induce 3xFlag-Sp5. C. Luciferase assays of *Wnt3a^DE^*-luc deletion constructs. Schematic depicts the relative locations of Sp5, Cdx2, Tcf/Lef, and T-box binding motifs in the deletion constructs. D. (Top) Schematic showing polarized *Wnt3a* expression (green) during gastruloid elongation. (Bottom) HCR analysis of *Wnt3a* (green), *Tbxt* (red), and *Cdx2* (cyan) expression in WT(R1) and *Wnt3a^ΔDE^* gastruloids one (D4) and two (D5) days after CHIR treatment, respectively. Dapi, grey.

Functional characterization of the *Wnt3a*^*DE*^ (Enhancer E1) and E2 elements (Fig. 5A) in luciferase reporter assays in differentiating ESCs confirmed that the *Wnt3a*^*DE*^, but not the E2 element, could drive transcription in response to CHIR and bFGF treatment (Fig. 6A). Combining the E2 element with *Wnt3a*^*DE*^ reduced the activity of *Wnt3a*^*DE*^ by 10-fold suggesting that E2 possesses silencer activity. Overexpression of Flag-Sp5 further enhanced transcriptional activity of the *Wnt3a*^DE^ element when stimulated by CHIR alone or together with bFgf (Fig. 6B). Enhancer deletion experiments showed that the Sp and Cdx sites are necessary but not sufficient for enhancer activity (Fig. 6C). Similarly, Tcf/Lef and Tbxt binding sites are necessary but insufficient for enhancer activity suggesting that all four TFs are required for robust *Wnt3a*^DE^ activity.

To determine if *Wnt3a*^*DE*^ is necessary for *Wnt3a* expression during NMC formation and axial elongation, we deleted the element in ESCs using CRISPR/Cas9 gene editing (Supp. Fig. 6A) and then examined the consequences of this mutation on gastruloid development. Gastruloids are self-organizing three-dimensional ESC aggregates that undergo morphogenesis in vitro characteristic of embryo axial elongation (Beccari et al., 2018; van den Brink et al., 2020; Veenvliet et al., 2020). A brief treatment of epiblast-like aggregates with CHIR initiates posterior polarization and PS differentiation programs that establish the Wnt3a-Tbxt feedback loop and leads to self-organization and axial elongation. HCR WISH demonstrated that posterior-polarized domains of *Wnt3a, Tbxt* and *Cdx2* co-expression were apparent in control gastruloids by D4 and highly expressed by D5 due to positive feedback (Fig. 6D). In contrast, gastruloids lacking the *Wnt3a*^DE^ did not express *Wnt3a* on D4 and only expressed *Tbxt* and *Cdx2* at low levels (Fig. 6D, right). *Tbxt* and *Cdx2* were undetectable by D5. Thus, the *Wnt3a*^*DE*^ is necessary for *Wnt3a* expression and to sustain the Sp5/8-Tcf7-Cdx-Tbxt autoregulatory loop that maintains NMCs.

### Sp5 regulates Wnt target gene expression by controlling Tcf7 and Tle occupancy at WREs

**Fig.7:**
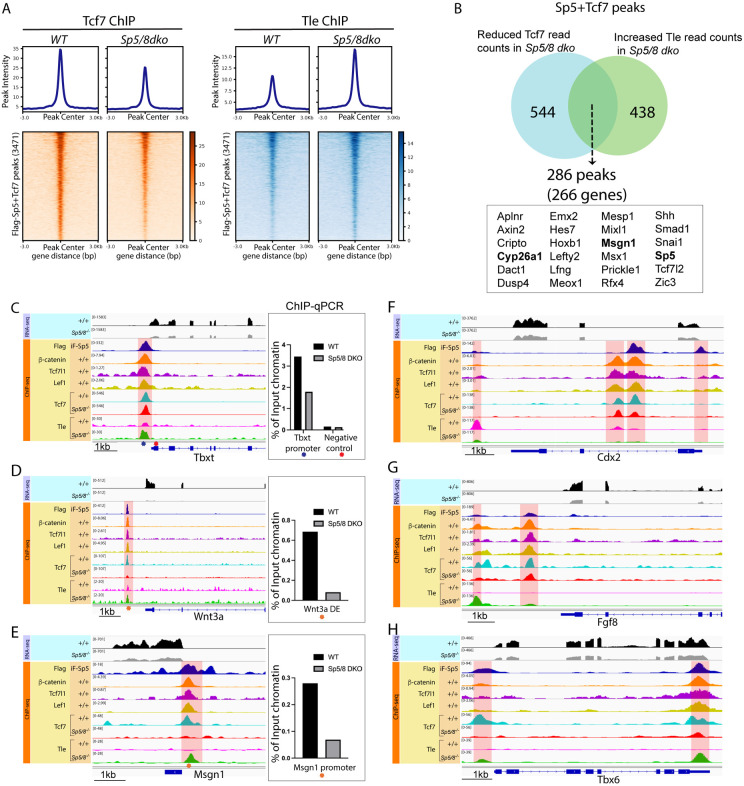
Sp5/8 regulate Tcf7 and Tle occupancy at WREs. A. Metaplots and heatmaps of Tcf7 and Tle binding profiles at sites of Sp5 and Tcf7 co-occupancy in WT and *Sp5/8* dko NMCs. B. Identification of potential sites of Sp5/8-dependent Tcf exchange. 286 WREs defined by Sp5 and Tcf7 co-occupancy in wt cells display reduced Tcf7, and increased Tle, peak intensities in *Sp5/8* dko cells. Only peaks with a log2 fold change > 0.58 and a P-value ≤ 0.05 were included in this analysis. C. IGV genome tracks of RNA-seq and ChIPseq at select Wnt target genes in +/+ and *Sp5/8^−/−^* (dko) mutants. Top rows in each set depict RNAseq expression data obtained from E8.5 +/+ and *Sp5/8^−/−^* embryos. Bottom rows illustrate ChIP-seq data for Flag-Sp5 (in iF-Sp5 cells), Tcf7 and Tle performed in +/+ and *Sp5/8^−/−^* EBs. β-catenin, Tcf7l1, Lef1 ChIP data from (Blassberg et al., 2022). *Tbxt* (a), *Wnt3a* (b), *Msgn1* (c), *Cdx2* (d), *Fgf8* (e) and *Tbx6* (f) loci are shown. ChIP-qPCR is shown for *Tbxt, Wnt3a*, and *Msgn1*.

We have previously shown protein-protein interactions between Sp5/8 and Tcf7/Lef1, however the functional relevance of this interaction for Tcf7/Lef1 activity and Wnt target gene transcription remains unclear (Kennedy et al., 2016). To determine whether Sp5/8 regulate Tcf7 binding at WREs, we performed ChIP-seq for Tcf7 and Flag-Sp5 in CHIR-stimulated embryoid bodies (EBs) generated from WT or *Sp5/8* dko ESCs (Supp. Fig. 6B, C). 28,154 Tcf7 peaks were identified (Supp. Fig. 7A, Supp. data file-5). Interestingly, the average Tcf7 peak intensity value, which is a measure of read density at a specific genomic region, was reduced by half in *Sp5/8* dko cells, suggesting that Sp TFs regulate Tcf7 binding. Examination of genomic regions that bound both Tcf7 and Flag-Sp5 identified 3471 sites, corresponding to 1168 genes, that were co-occupied by both TFs (Supp. Fig. 7B). The average Tcf7 peak intensity value at these putative WREs was considerably higher (35) than that observed for genomic Tcf7 binding sites without a demonstrated Sp5 peak (17) (cf. Fig. 7A and Supp. Fig. 7A) suggesting that the presence of Sp5 enhanced the binding of Tcf7 to DNA. This is supported by the observation that Tcf7 binding strength at these co-occupied sites was reduced by ~30% in *Sp5/8* dko cells (Fig. 7A). This reduction in Tcf7 binding was not due to changes in Tcf7 expression as Tcf7 protein and RNA levels did not depend on Sp5/8 levels (Supp. Fig. 7C, D). We then asked whether these changes in Tcf7 binding activity correlated with changes in Wnt target gene transcription. Of the 1168 Tcf7-Sp5 co-bound genes, 81 were downregulated in *Sp5/8* dko embryos, including the well-characterized Wnt/Sp target genes *Cdx1,2, Msgn1, Tbxt* and *Tbx6* (Supp. Fig. 7B). Together, our findings suggest that Sp5/8 modulate Wnt target gene transcription by enhancing Tcf7 occupancy at WREs.

The “Tcf exchange” model proposes that WREs are bound by repressive Tcf-Tle complexes when Wnt target gene expression is reduced, and that they are exchanged for activating Tcf7/Lef1-β-catenin complexes when target genes are active (Guo et al., 2021; Ramakrishnan and Cadigan, 2017). To examine the genomic distribution of repressive Tcf-Tle complexes in the presence or absence of Sp5/8, a pan-Tle antibody was chosen for ChIP as it detected Tle1-4 regardless of their Tcf partners. We then asked if Tle1-4 could be detected at the 3471 Tcf7-Sp5 co-bound WREs. Small Tle peaks were identified at these WREs in WT cells demonstrating that both repressive Tle and activating Tcf7 could be detected at active genes (Fig. 7A). Intriguingly, whereas Tcf7 peak intensity was reduced at WREs in *Sp5/8* dko cells, Tle peak intensities nearly doubled (Fig. 7A, Supp. Fig. 7A). qPCR and Western blot experiments suggested that increased Tle1-4 binding was not due to elevated Tle1-4 expression since no significant changes in protein or RNA levels were detected in the presence or absence of Sp5/8 (Supp. Fig. 7C, D). Rather, MEME-ChIP TF motif analysis suggests a striking change in the TFs that Tle1-4 associated with when *Sp5/8* were mutated. While Tcf/Lef motifs were not associated with Tle1-4 binding sites in CHIR-stimulated WT EBs, Tcf/Lef and Tbx motifs, amongst others, were increasingly associated with Tle binding peaks in *Sp5/8* dko EBs (Supp. Fig. 8A, B). These findings suggest that the reduced expression of Wnt target genes in *Sp5/8* dko cells correlates with an exchange of activating Tcf7-β-catenin for repressive Tle-Tcf at WREs. Gene ontology and KEGG pathway analysis of the 266 genes that displayed both reduced Tcf7 peak intensities and increased Tle peaks in *Sp5/8* dko cells compared to WT cells confirmed that these genes are associated with Wnt signaling, stem cells, gastrulation and AP patterning and include *Cyp26a1, Msgn1,* and *Sp5* (Fig. 7B, Supp. Fig. 8C, 8D, Supp. data file-6).

Detailed assessments of ChIP genome tracks at major regulators of axial progenitors for which there are well-characterized WREs, specifically *Tbxt, Wnt3a, Cdx2, Msgn1, Fgf8* and *Tbx6* (Amblard et al., 2024; Chalamalasetty et al., 2011; Nowotschin et al., 2012; Yamaguchi et al., 1999) showed that these WREs are bound by multiple Wnt/ β-cat transcriptional regulators ie. Sp5, β-catenin, Tcf7l1, Tle, Lef1 and Tcf7 (Fig. 7C-H). Consistent with the genome-wide analysis, the Tcf7 peaks were reduced in intensity at 5 of 6 of these NMC genes in *Sp5/8dko* EBs (Fig. 7D-H). The loss of Tcf7 binding in *Sp5/8* dko mutants was most notable at the *Wnt3a*^*DE*^ and the *Msgn1* promoter, and this correlated well with the dramatic reductions in transcript levels (Fig. 7D, E). ChIP-qPCR validated the ChIPseq results for *Wnt3a* and *Msgn1*. Interestingly, despite our inability to observe differential binding of Tcf7 at the *Tbxt* promoter by ChIPseq, ChIP-qPCR detected reduced Tcf7 binding in *Sp5/8* dko EBs (Fig. 7C). In contrast to the reduced Tcf7 binding, elevated Tle peaks were observed at the corresponding WRE for 5 of 6 genes in *Sp5/8* mutant cells (Fig. 7C-E, G, H). Together, these results suggest that Sp5 enhances Tcf7, and reduces Tle, occupancy at the WREs of active Wnt target genes. The absence of Sp5-Tcf7 interactions in *Sp5/8* mutants leads to a Tle-Tcf substitution that represses Wnt target gene expression.

## Discussion

### Sp5/8 are integral components of the Wnt3a-Tbxt feedback loop in axial progenitors

Our findings demonstrate that Sp TFs are essential regulators of axial progenitor homeostasis, functioning to control morphogen signaling and TF networks within the NMC niche. Tissue-specific deletion of *Sp5* and *Sp8* in the PS resulted in a severe axial truncation due to the depletion of trunk axial progenitors. Notably, the anterior positioning of the axial truncation in *Sp5/8* double mutants, compared to *Sp8* single mutants which display a more posteriorly positioned tail truncation (Bell et al., 2003; Harrison et al., 2000), suggests a dose-dependent requirement for Sp5/8 in axial elongation that closely resembles previously described dose-dependent phenotypes in the Wnt pathway ex. *Wnt3a;8a, Wnt3a;vestigial tail, Tcf1;Lef1,* and *Cdx1;2* double mutants, and Cdx2; *Tbxt* compound mutants (Chawengsaksophak et al., 2004; Chawengsaksophak et al., 1997; Galceran et al., 1999; Greco et al., 1996; MacMurray and Shin, 1988; Savory et al., 2009; Stott et al., 1993; van den Akker et al., 2002; van Nes et al., 2006; Young et al., 2009). Genetic interactions between *Sp5* and *Wnt3a* (Dunty et al., 2014), *Sp5* and *Tbxt* (Harrison et al., 2000), and *Tbxt* and *Cdx2* (Amin et al., 2016) firmly place *Sp5* and, by extension, *Sp8,* in this genetic *Wnt3a, Tcf1/Lef1, Tbxt* and *Cdx2* pathway. We suggest that increasing activity of this canonical Wnt/β-cat pathway is necessary for the emergence of increasingly posterior progenitors.

Axial progenitors must persist in the CLE and PS and, later, in the tailbud for at least 5 days of embryonic development (E7.5-12.5) to generate the complete trunk and tail (Cambray and Wilson, 2002, 2007). As axial development progresses in an anterior-to-posterior direction, early disruptions in progenitor maintenance result in anterior truncations, while later disruptions cause posterior truncations. The anterior truncation observed in *Sp5/8* double mutants aligns with an early requirement in trunk progenitors while the *Sp8* single mutant phenotype aligns with a later requirement in tail progenitors. A plausible interpretation of the correlation between axial position and *Wnt3a* or *Sp5/8* gene dosage is that increasing Wnt activity is required to sustain axial progenitor self-renewal over time. In other words, Wnt/Sp signaling regulates the orderly timing of posterior progenitor emergence. The higher the Wnt/Sp signal, the later posterior progenitors emerge from the PS. This is supported by gain of function studies in which overexpression of Sp8 in axial progenitors led to the retention of axial progenitors in the caudal progenitor zone. We suggest that elevated levels of Sp propagate the Wnt3a-Tbxt-Cdx feedback loop for longer, thereby maintaining the self-renewal of increasingly posteriorized progenitors, possibly through Cdx and the maintenance of the Hox clock (Deschamps and Duboule, 2017).

The identification of Sp5 and Sp8 as both downstream targets and upstream regulators of Wnt3a, Tbxt and Cdx2 strongly suggests their participation in an autoregulatory loop essential for axial progenitor maintenance. Previous work identified *Tbxt* as a direct target of Wnt3a/β-cat signaling (Arnold et al., 2000; Yamaguchi et al., 1999), but the mechanisms governing *Wnt3a* activation by Tbxt remained elusive. Our data now reveal that Tbxt, Sp5, Sp8, Cdx2, β-catenin and Tcf7 converge on a conserved enhancer downstream of *Wnt3a* to form a TF complex that regulates enhancer accessibility and gene expression. Single-cell ATAC-seq analysis in *Tbxt* mutants confirmed a crucial role for Tbxt in maintaining chromatin accessibility at this enhancer, reflecting a broader role of Tbxt in regulating the axial progenitor gene program (Koch et al., 2017). Examination of *Wnt3a, Fgf4, Fgf8, Sp5, Sp8, Lef1, Tbxt, Cdx1, 2* and 4 reveals the regulatory logic that Tbxt and associated TFs use to activate these axial progenitor genes - they are bound by Tbxt, Tcf7, and either Cdx2, Sp5 or Sp8 (this work; (Amin et al., 2016; Kennedy et al., 2016; Koch et al., 2017; Savory et al., 2009; Zhu and Lohnes, 2022). We propose that reciprocal interactions among Sp5, Sp8, Tcf7, Tbxt and Cdx TFs constitute a robust transcriptional module that collectively sustains *Wnt3a, Fgf4* and *Fgf8* expression in axial progenitors through positive feedback.

### Sp5/8 and the NMC niche

A stem cell niche is defined as a specialized microenvironment that maintains progenitor self-renewal. The niche contains support cells that provide a local source of signals that regulate stem cell homeostasis. In adult intestinal stem cells (ISC), niche signals such as Wnt3a are secreted by neighboring epithelial Paneth cells (in humans) and underlying mesenchymal stromal cells (Clevers et al., 2014; Santos et al., 2018). By analogy, the self-renewal of embryonic NMCs relies on Wnt3a, however, the cellular composition of the NMC niche is inadequately defined. Our results strongly suggest that the PS itself acts as a critical signaling center in the niche. Axial progenitors residing in the CLE are likely regulated by both autocrine and paracrine signaling as *Wnt3a, Wnt8a, Fgf4, Fgf8* and *Fgf17* are expressed in NMCs and, more broadly, throughout the PS (Cunningham et al., 2015; Dunty et al., 2008; Dunty et al., 2014; Gouti et al., 2017; Koch et al., 2017; Maruoka et al., 1998; Pijuan-Sala et al., 2019; Tran et al., 2024; Wahl et al., 2007). Together with the high levels of Wnt and Fgf emanating from the posteriorly-positioned PS, low levels of RA secreted from anteriorly-positioned somites define a signaling environment that maintains NMCs (Gouti et al., 2017). Reduced expression of the RA-metabolizing enzyme *Cyp26a1* in *Sp5/8 dko* axial progenitors demonstrates that Sp5 and Sp8 also regulate RA levels in the NMC niche (Supp. Fig. 4). We conclude that Sp5/8 play a central role in the NMC niche to establish a high Wnt/Fgf, low RA signaling environment that ensures spatially coordinated neural and mesodermal differentiation.

The node has also been implicated as a niche component that sustains NMCs through paracrine signals (Wymeersch et al., 2019). The node largely consists of notochord progenitors and lies ventral to, and in direct contact with, NMCs throughout axial elongation. Localized ablation of ventral node cells at the NSB at E8.5 perturbed axis elongation, leading to speculation that the crown cells of the node provide signals that sustain NMPs. We used conditional, tissue-specific targeting of the PS as well as null alleles to investigate the role of Sp5/8 in axial progenitors. The observed expression of Sp5/8 in the node raises the intriguing possibility of their involvement in node signaling. Given that the E8.5 node is ciliated and is a powerful organizer of the Left-Right axis, future studies will attempt to discern potential roles for Sp5/8 in the node. We suggest that the NMC niche is composed of two neighboring but spatially distinct support cell populations, the PS, lying caudal to NMCs, and the node lying immediately ventral.

### Sp5/8 and Wnt target gene regulation

Given the crucial roles that the canonical Wnt/β-cat pathway plays in the regulation of stem cells and in the etiology of human diseases such as cancer, it is of fundamental importance to understand how the pathway regulates target gene transcription. Most target genes of the Wnt/β-catenin pathway are regulated by the Tcf TFs, of which there are 4 in vertebrates – Tcf7(Tcf1), Lef1, Tcf7l1(Tcf3) and Tcf7l2(Tcf4). Molecular genetic studies suggest that vertebrate Tcfs function predominantly as dedicated activators (Lef1), repressors (Tcf7l1) or both (Tcf7 and Tcf7l2) depending on the context (reviewed in (Bou-Rouphael and Durand, 2021; Guo et al., 2021; Ramakrishnan et al., 2018)). Repressor activity strongly correlates with their affinity for Tle co-repressors as the dedicated repressor Tcf7l1 binds strongly to Tle while the activator Lef1 does not (Chodaparambil et al., 2014). While it is oft-stated that Tcfs can be converted from repressors to activators by the β-catenin-mediated displacement of Tle from Tcf, β-catenin and Tle1 can bind simultaneously to Tcf7l1 suggesting that they do not simply compete for binding to Tcf (Chodaparambil et al., 2014). The precise mechanisms controlling the transcriptional off/on switch at Wnt target genes remains an open question. Only one Tcf (Pangolin) exists in Drosophila, necessitating a transcriptional switch model in which Pangolin functions as both repressor and activator depending on its bound cofactor – repressive Groucho or activating β-catenin. However, the existence of specialized vertebrate Tcfs have led to the development of the “Tcf exchange” model where, for example, repressive Tcf7l1-Tle complexes are replaced by activating Tcf7-β-catenin complexes. How active Wnt target gene expression is terminated is also not well-understood but presumably requires continued exchange of activating Tcf complexes for repressive ones.

Our ChIP studies investigating the genomic distribution of Sp5/8 binding sites show that Sp’s are bound to many of the same genomic sites as the Wnt transcriptional effectors β-catenin, Tcf7, Lef1, Tcf7l1 and Tle1-4. Given that Sp5 can bind directly to Tcf7 and Lef1 (Kennedy et al., 2016), these results demonstrate that Sp TFs are physically connected to at least two terminal effectors of Wnt/β-catenin signaling. For many of the target genes that we have focused on in this work, these genomic sites are well-characterized WREs, functioning as cis-regulatory elements to drive tissue-specific gene expression in stem cells and in the embryo. In addition to binding Sp5/8 and Wnt effectors, these WREs are also bound by Tbxt and Cdx TFs which, together with Sp5/8, constitute a core autoregulatory circuit that drives the self-renewal of axial progenitors. Thus Sp5/8 function to link the canonical Wnt/β-catenin signaling pathway to the core regulatory circuitry of axial stem cells.

**Fig.8.**
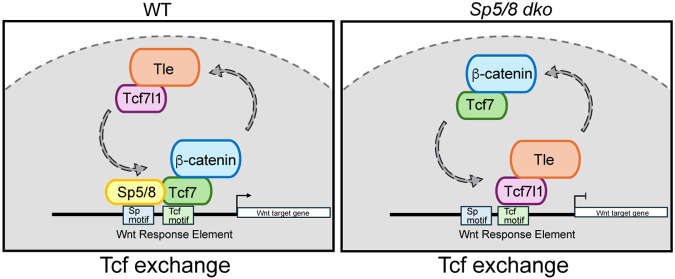
Model: Sp5/8 regulate Tcf exchange to promote Wnt target gene transcription. *Sp5* is induced by Wnt ligand. Elevated levels of Sp5 promote the dynamic exchange of repressive Tcf7l1-Tle complexes for activating Tcf7-β-catenin complexes at Wnt Response Elements (WREs). We speculate that the binding of Sp5/8 to DNA and to Tcf7 enhances the activation of Wnt target genes by modulating Tcf7-β-catenin binding kinetics at WREs. In the absence of *Sp5/8*, repressive Tcf7l1-Tle replace activating Tcf7-β-catenin complexes, resulting in the down regulation of Wnt target gene expression.

We have presented evidence that Sp5/8 promote Tcf7 occupancy at WREs of actively transcribed genes. Tcf7 peak binding intensities are reduced at WREs in the absence of Sp5/8 activity, but Tcf7 continues to bind to canonical Tcf motifs (Supp. Fig. 8A) suggesting that the binding of Sp5/8 to Tcf7 does not alter Tcf7 binding specificity. Interestingly, these sites are also bound by negative regulators of Wnt target gene transcription including Tcf7l1 and Tle1-4. Thus, both positive and negative regulators may be bound at any time and the transcriptional output may be determined combinatorially and by the predominance of a given regulator. Considering that the ChIPseq is performed on populations of cells, it is also possible that positive and negative regulators may be bound at a given WRE in distinct cells. Nevertheless, our demonstration that Tle binding increases, when Tcf7 decreases, at many WREs in *Sp5/8* mutant cells, is consistent with the Tcf exchange model (Fig. 8) however a limitation of our work is that we did not specifically address the genomic distribution of Tcf7l1. It is also worth noting that while dramatic reductions in Tcf7 binding were noted at *Wnt3a, Msgn1, Tbx6, Cdx2* and *Fgf8,* changes in Tcf7 peak intensity were not consistently observed at the *Tbxt* WRE in *Sp5/8* mutants despite the clearly elevated levels of Tle observed there. This leaves open the possibility that a binary transcriptional switch is in effect at *Tbxt,* in which Tcf7 remains bound at the WRE, despite the absence of Sp5/8, and β-catenin is replaced by Tle to convert Tcf7 to a repressor.

Our results suggest that a role for Sp5 in the Wnt/β-catenin pathway is highly conserved, functioning in vertebrates and invertebrates to regulate trunk and tail fates (Dailey et al., 2017; Dunty et al., 2014; Gautam et al., 2024; Mohanty and Lekven, 2025; Tewari et al., 2019; Weidinger et al., 2005). Interestingly, Sp TFs display context-dependent functions, acting as transcriptional activators or repressors depending on tissue and developmental stage-specific partners. We have shown that Sp5/8 bind directly to Tcf7 and Lef1 to stabilize their binding to a *Wnt3a* enhancer to establish a positive feedback loop (this work; (Kennedy et al., 2016). Abundant evidence shows that Sp5 can also function as a feedback repressor during AP development. For example, in the regenerating acoel, Hofstenia miamia, a small group of Wnt-dependent genes, including *Sp5, Tbxt* and *Hox* genes, are expressed in the tail where Sp5 acts to repress Wnt-regulated trunk genes (Tewari et al., 2019). In *Hydra,* Sp5 represses *Wnt3* to establish a negative feedback loop that suppresses ectopic head formation (Vogg et al., 2019). Additionally, Sp5 represses a Wnt-dependent transcriptional program in differentiating human PSCs (Huggins et al., 2017). Future studies will clarify how distinct co-factors could modulate Sp activity in diverse developmental contexts. Taken together, our work supports a conserved role for Sp’s in Wnt-dependent autoregulatory loops and highlights the importance of feedback control in the precise regulation of Wnt signaling in stem cells.

## Materials and Methods

### Mouse strains and skeletal preparation

*Sp5*^*lacZ/lacZ*^*; Sp8*^*+/Δ*^*, T-Cre, Sp8* ^*GOF*^*(TetO-Sp8 Ires-EGFP), Rosa26-rtTA-IRES-EGFP* (B6.Cg-*Gt(ROSA)26Sor*^*tm1(rtTA,EGFP)Nagy*^/J) mice were described previously (Dunty et al., 2014; Kennedy et al., 2016; Perantoni et al., 2005). All mice were staged by somite number. *Sp8*^*GOF*^ mice were crossed with *Rosa26-flox-rtTA* mice to enable temporal and conditional expression of Sp8 using the *T-Cre* line. For temporal expression, pregnant females were provided with Dox chow (200mg/kg) (Bio-Serv, S3888) and Dox water (1.6mg/ml wt/vol Dox) in 5% (wt/vol) sucrose from embryonic day 7.5 (E7.5) and were harvested at embryonic day 8.5 (E8.5). All mouse experiments were conducted in accordance with the animal protocol approved by the NCI-Frederick Animal Care and Use Committee. Skeletal preparations of E18.5 fetuses were done as described previously (Hogan and Hogan, 1994).

### Whole mount in situ hybridization

For colorimetric whole mount in situ hybridization (WISH) experiments, Dig-labeled probes were synthesized, and WISH was conducted as described (Dunty et al., 2008). For multiplexing whole mount Fluorescent In Situ Hybridization (FISH), split initiator hairpin probes (V 3.0) were custom-designed and synthesized by Molecular Instruments, Inc. Buffers and amplifiers conjugated to fluorophores were procured from Molecular Instruments, Inc, and FISH was carried out according to the manufacturer’s instructions (Choi et al., 2018). Embryos and gastruloids were harvested and fixed in 4% PFA overnight at 4°C and dehydrated in Methanol/PBST (PBS+0.1%Tween-20) series the next day. For HCR hybridization, samples were rehydrated in Methanol/PBST series and treated with Proteinase-K for 15 min followed by post-fixation in 4% PFA for 20 min at room temperature. After pre-incubation with probe hybridization buffer, samples were incubated with probe mix at 37°C for overnight. Next day, samples were washed, and amplification was performed in 60 pmol of each hairpin in 0.5 ml of amplification buffer overnight at room temperature. Next day, samples were washed in 5x SSCT buffer and stained in 0.5 ug/ml Dapi in 5x SSCT buffer overnight in 5x SSCT, then embedded on glass bottom dishes (MatTek or Greiner Bio-One GmbH) in 1% ultra-low melt agarose (Cambrex). Subsequently, samples were cleared at room temperature using Ce3D++ solution for 3-4 days before confocal imaging, as described in (Anderson et al., 2020). Whole mount FISH was performed similarly for gastruloids, with the exception that gastruloids were not rotated on a shaker.

### Whole mount immunohistochemistry

For Whole Mount Immunohistochemistry (WIHC), embryos were fixed for 30 minutes in 4% paraformaldehyde at room temperature and subsequently washed in PBST (PBS+0.1% Tween-20). Permeabilization was achieved using 0.5% Triton-X 100, followed by blocking in 5% normal donkey serum in PBST for 1 hour. Embryos were then incubated in 100 μL of primary antibodies overnight at 4°C with shaking, followed by 5-6 PBST washes over 24 hours. Subsequently, embryos were incubated in Alexa Fluorophore-conjugated secondary antibodies (1:2000) at 4°C on a shaker, followed by another day of PBST washes containing 0.5 μg/mL DAPI. Finally, embryos were mounted as described above. Antibody information can be found in Supp. data file 7.

### scRNA-seq and expression heat maps

UMAP projection plots for E8.25 embryo set for *T/Bra, Sox2, Sp5,* and *Sp8* were extracted from MouseGastrulationData R/Bioconductor package (v1.20.0) https://marionilab.cruk.cam.ac.uk/ (Pijuan-Sala et al., 2019). Heatmaps for expression of *T/Bra, Sox2, Sp5,* and *Sp8* from different treatment conditions in differentiating ESCs were extracted from multiplexed Barcodelet Single-Cell RNA-seq (Yeo et al., 2020).

### Bulk RNA-seq

For Bulk-RNA sequencing, embryos were collected at 5-7 somite stages, bisected between somites (S)1 and S2, and posterior fragments were collected for RNA isolation. Bulk RNA-seq paired end reads were processed using the REN EE v2.5.8 (Rna sEquencing aNalysis pipElinE) analysis pipeline (https://github.com/CCBR/renee) using default settings. Briefly, low quality reads and adaptors were removed using Cutadapt v1.18 (Martin, 2011) before undergoing alignment to the GRCm38.p6 genome using STAR v2.7.6a in two-pass mode (Dobin et al., 2013). Post-alignment QC metrics were collected using Picard v 2.17.11 and RSeQC v 2.6.4 while potential contamination was examined using the Kraken v 2.0.8-beta and FastQ Screen v 0.14.0 packages (Wang et al., 2012; Wingett and Andrews, 2018; Wood and Salzberg, 2014). Expected and isoform counts, according to the GENCODE vM25 primary assembly annotation reference, were collected using the RSEM v1.3.0 package. Differential expression analysis was performed using DESeq2 v1.42.0 in R v4.3.0 (Love et al., 2014; Soneson et al., 2015). Genes with less than 10 counts in at least 3 samples were filtered out. Genes were considered differentially expressed if they presented a log 2-fold change > 0.5 or < −0.5 and an adjusted P-value < 0.05 (Benjamini-Hochberg). The cluster Profiler v4.10.0 R/Bioconductor package was used to evaluate enrichment of Gene Ontology biological processes terms associated with lists of differentially expressed genes through Gene Set Enrichment Analysis (adjusted P-value < 0.01) (Yu et al., 2012). We used the rrvgo package to reduce and interpret the list of associated GOs obtained from cluster Profiler (Sayols, 2023). For that we first defined two sets of GOs associated with either overexpressed or under expressed genes using their normalized enrichment scores. Each GO set was used to compute a similarity matrix considering the full set of mouse genes as background. We proceed by computing the scores of each GO term, defined as the −log10 of their adjusted p-values, and used the similarity matrices, together with the list of scores, to obtain two lists of reduced terms (threshold of 0.7). Enhanced Volcano and Complex Heatmap and Venn Diagram packages were used for visualization throughout. For integrating published data sets, differentially expressed genes in the bulk-RNA seq for *T^2J/2J^* embryos (Koch et al., 2017) and *Cdx* triple knockout embryos (Amin et al., 2016) were used. Raw and processed sequencing files have been deposited at the GEO database.

### ChIP-seq

ChIP-seq experiments analysing Flag-Sp5 genome occupancy was described previously (Kennedy et al., 2016). ChIP-seq experiments for Tbxt, Flag-Sp5 and Flag-Sp8 were performed in A2lox.Cre ESCs (Iacovino et al., 2011), while Tle and Tcf7 ChIPs were conducted in R1 ESCs. ESCs were differentiated into NMCs as described previously (Gouti et al., 2014) in 2 dimensional cultures or embryoid bodies. Pluripotent ESCs were differentiated with bFGF (12ng/ml) for 2 days (D0-D2) followed by 3uM CHIR and 12ng/ml bFGF for 24 hours. For overexpression of inducible Flag-Sp5, 1ug/mL doxycycline (dox) was added from D2 to D3. Cells were harvested and fixed in 1% formaldehyde solution for 15min, quenched in 0.125M glycine and flash frozen. For chromatin immunoprecipitation and sequencing, nuclear extracts were incubated with antibodies as listed in Supp. data file 7. ChIP libraries were prepared and sequenced either on two separate NextSeq runs using V2 chemistry or on the NovaSeq Xplus 1.5B platform utilizing the IDT 2S Plus DNA library preparation kit (SWIDT) with single-end sequencing. All libraries demonstrated high sequencing quality, with over 94% of bases exceeding a Q30 quality score. Sequencing yield ranged from 42 to 67 million pass-filter reads per sample, indicating robust library preparation and sequencing performance. Downstream data processing and analysis were conducted using the CHAMPAGNE pipeline (DOI: 10.5281/zenodo.10516078), a comprehensive and reproducible workflow developed for ChIP-seq data analysis. Raw reads were first subjected to adapter trimming using Cutadapt (Martin, 2011), followed by library complexity estimation using Preseq, enabling projection of sequencing diversity and detection saturation. For peak detection, MACS2 (Zhang et al., 2008) was employed with merged input controls generated by aggregating input replicates corresponding to each immunoprecipitation (IP) group. To ensure consistency and reproducibility across biological replicates, replicate concordance was assessed using bamCompare from the deepTools suite (Ramirez et al., 2016). Further, deepTools was also utilized to generate normalized heatmaps depicting read pileups surrounding peak regions, providing insight into binding enrichment and distribution. Differential binding analysis was conducted using DESeq2 and ChIP-seq peaks with log2 Fold change >0.58 or <−0.58 and *P*-value ≤ 0.05 were considered significant for further analysis. For gene set Over Representation Analysis (ORA) for GO biological processes and significant KEGG pathways, WEB-based GEne SeT Analysis Toolkit was used (Elizarraras et al., 2024).

### Nuclear extracts and western blots

For nuclear protein extracts, nuclei were isolated using Buffer A [10 mM Hepes, 1.5 mM MgCl_2_, 10 mM KCl], followed by nuclear disruption in Hepes lysis buffer [50 mM Hepes (pH 7.9), 150 mM NaCl, 0.5% Triton]. Both buffers were supplemented with EDTA-free protease inhibitors. The lysates were resuspended in 1× Laemmli buffer for subsequent immunoblotting. Lysates were loaded onto a 4-12% Bis-Tris gel and transferred to a PVDF membrane using the eBlot^™^ L1 fast wet transfer system (GenScript). The transferred proteins were incubated with blocking buffer (TBS [pH 8], 0.1% Tween 20, and 5% non-fat dry milk) for 1 hour at room temperature, followed by overnight immunoblotting with primary antibodies (see Supp. data file 7) at 4°C. Protein detection was performed using SuperSignal West Pico PLUS Chemiluminescent Substrates (Protein Biology, 34577).

### Confocal imaging and image processing

Whole mount colorimetric in situ embryos were imaged on a Zeiss Axioplan2 microscope. For whole mount FISH experiments, Ce3D++ was removed and confocal images were acquired on a Nikon A1R microscope using a 10x objective; NA = 0.4 and 3.3μm Z-sections. For midline sagittal sections of embryos processed for whole mount immunohistochemistry (see Fig. 4B), the posterior third of the embryo was dissected, flatmounted and scanned from ventral to dorsal using a 25x silicon objective; NA=1.0 and 0.2μm Z-sections. All images were denoised and sharpened using NIS elements software (Nikon) and image projections were analyzed using Fiji (25) or Imaris software (v 9.8-10.0.1, Oxford Instruments). For optical cross sections through the primitive streak, the posterior third of the embryo was embedded vertically in 1% agarose on a glass bottom dish and confocal scanning was performed from posterior to anterior at 1μm Z-sections. A minimum of 3 embryos and 5 gastruloids were analyzed for each genotype per probe.

### Embryonic stem cell culture and gastruloid assay

ESCs were routinely cultured on mouse embryonic fibroblasts or feeder-free 0.1% gelatin coated dishes as described previously (Kennedy et al., 2016). Serum and feeder-free ESCs were maintained in N2B27 media supplemented with LIF, CHIR99021, and PD0325901 (2i+LIF) on gelatin coated dishes. Before gastruloid experiments were initiated, ESCs were passaged at least 5 times in GMEM/10% FBS supplemented with non-essential amino acids, Glutamax, Sodium pyruvate, beta-mercaptoethanol and LIF as described previously (Beccari et al., 2018). ESCs were grown to 60-70% confluency and split using TrypLE select (Thermofisher Scientific, 12563029). Cells were washed twice with warm PBS (without CaCl2 and MgCl2) and resuspended in NDiff227 (Takara, Y40002) or ESGRO complete basal media (Millipore Sigma, SF002-500). Gastruloid formation was initiated with 300 cells/well in 96 well ultra-low attachment dishes (Corning, 3474). After 2 days, aggregates were treated with 150μl of warm media supplemented with 1μM CHIR (Tocris, 4423). Pre-warmed media (37°C) was exchanged on D3 and D4, and gastruloids were harvested on D5 using a cut 1000μl tip and fixed overnight in 4% PFA followed by dehydration in a methanol series the next morning.

### Luciferase assays

330ng of luciferase and 5ng of Renilla constructs were co-transfected into wildtype R1 or A2Lox.Cre) ES cells on Day 1 of culture. Cells were treated with CHIR+bFGF from Day 2 to Day 3. Luciferase assays were performed according to Dual Luciferase Assay Kit (Promega) instructions.

### Gene targeting

To generate knockout cell lines, the nucleofection of CRISPR-Cas9/gRNA ribonucleoprotein complexes and pMax-GFP constructs was prepared with the Amaxa^™^ mouse ES cell nucleofector^™^ Kit (Roche, VPH-1001) following the manufacturer’s instructions. The gRNA sequences are described in Supp. data file 7. The nucleofection of Cas9/gRNA complex and pMax-GFP cocktail solutions into 2X10^6^ cells was performed using the Amaxa Nucleofector (Lonza). One day after nucleofection, GFP-positive cells were sorted using SH800 (Sony), and 3 X 10^5^ cells were plated onto a gelatin-coated 150mm dish. Colonies grown for 4-5days were picked and moved to a gelatin-coated 48 well plate. Genomic deletion was confirmed by Sanger sequencing (Psomagen, Rockville, MD).

## Supplementary Material

Supplement 1 Supplementary data file-1. Overlap of Sp5/8 bulk-RNA seq with Sp5 ChIP-seq (GSE73084) data. Related to Fig. 2C

Supplement 2 Supplementary data file-2. Overlap of *Sp5/8dko* and *Wnt3a ko* bulk RNA seq. Worksheets contain both unique, shared, and shared downregulated gene sets. Related to Fig.2D

Supplement 3 Supplementary data file-3. Comparative transcriptome analysis of *Sp5/8 dko, Wnt3a ko, Cdx tko, Tbxt ko* data sets. Related to Fig.3 and Supp. Fig. 2.

Supplement 4 Supplementary data file-4. Comparative ChIP-seq analysis of Flag-Sp5, Flag-Sp8, Cdx2 and Tbxt datasets. Related to Fig. 3.

Supplement 5 Supplementary data file-5. List of all ChIP-seq peaks for Flag-Sp5, Tcf7 and Tle conditions. Related to Fig. 7, and Supp. Fig. 7, 8.

Supplement 6 Supplementary data file-6. Summary of all ChIP-seq data comparisons. Related to Fig. 7, and Supp. Fig. 7, 8.

Supplement 7 Supplementary data file-7. Details of gRNAs, sequencing primers for characterizing knockout cells line, qPCR oligos, antibodies for Immunohistochemistry, ChIP-seq, Immunoprecipitations and western blots are included.

8
