# Supplementary material for: Sp Transcription Factors Establish the Signaling Environment in the Neuromesodermal Progenitor Niche During Axial Elongation
Source: bioRxiv. 2025 Jun 4:2025.06.03.657492. Preprint. [Version 1] doi: 10.1101/2025.06.03.657492 (PMC12157472; doi:10.1101/2025.06.03.657492)
Supplement: 8 [file NIHPP2025.06.03.657492v1-supplement-8.pdf]

bioRxiv preprint doi: https://doi.org/10.1101/2025.06.03.657492; this version posted June 4, 2025. The copyright holder for this preprint (which was not certified by peer review) is the author/funder. This article is a US Government work. It is not subject to copyright under 17 USC 105 and is also made available for use under a CC0 license.

Supplementary Figures

Supp. Fig.1

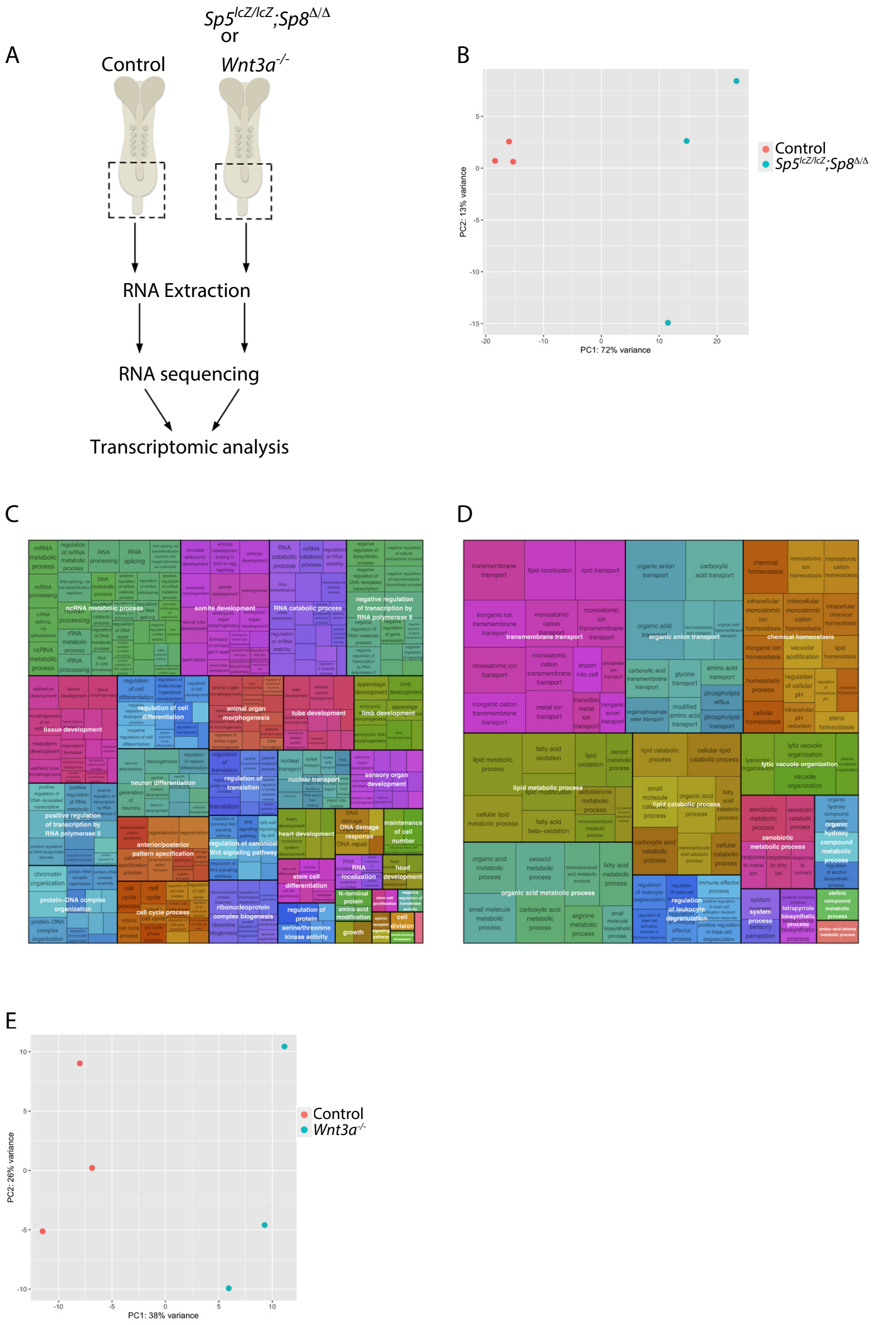

Supp. Fig.1. Gene Set Enrichment Analysis (GSEA) of differentially expressed genes in *Sp5/8* dko bulk-RNA seq. Related to Figs 1 and 2.

bioRxiv preprint doi: https://doi.org/10.1101/2025.06.03.657492; this version posted June 4, 2025. The copyright holder for this preprint (which was not certified by peer review) is the author/funder. This article is a US Government work. It is not subject to copyright under 17 USC 105 and is also made available for use under a CC0 license.

A. Schematic representation of E8.5 control and *Sp5/8 dko* or *Wnt3a*$^{-/-}$ mutants, with the dashed box illustrating the posterior termini dissected for RNA extraction and bulk-RNA sequencing (n=3 for controls and *Sp5/8 dko* mutants).

B. Principal component analysis of control (n=3) and *Sp5/8dko* (n=3) samples used for bulk-RNA seq analysis.

C-D. Treemap visualization of significantly enriched GO terms identified through GSEA analysis of *Sp5/8 dko* bulk RNA-seq differentially expressed downregulated genes (E) and upregulated genes (F). Each rectangle denotes a GO term, with similar terms grouped by color to reflect semantic similarity. Rectangle areas are proportional to adjusted p-values (-log10 transformed).

E. Principal component analysis of control (n=3) and *Wnt3a*$^{-/-}$ (n=3) samples used for bulk-RNA seq analysis.

bioRxiv preprint doi: https://doi.org/10.1101/2025.06.03.657492; this version posted June 4, 2025. The copyright holder for this preprint (which was not certified by peer review) is the author/funder. This article is a US Government work. It is not subject to copyright under 17 USC 105 and is also made available for use under a CC0 license.

Supp. Fig.2

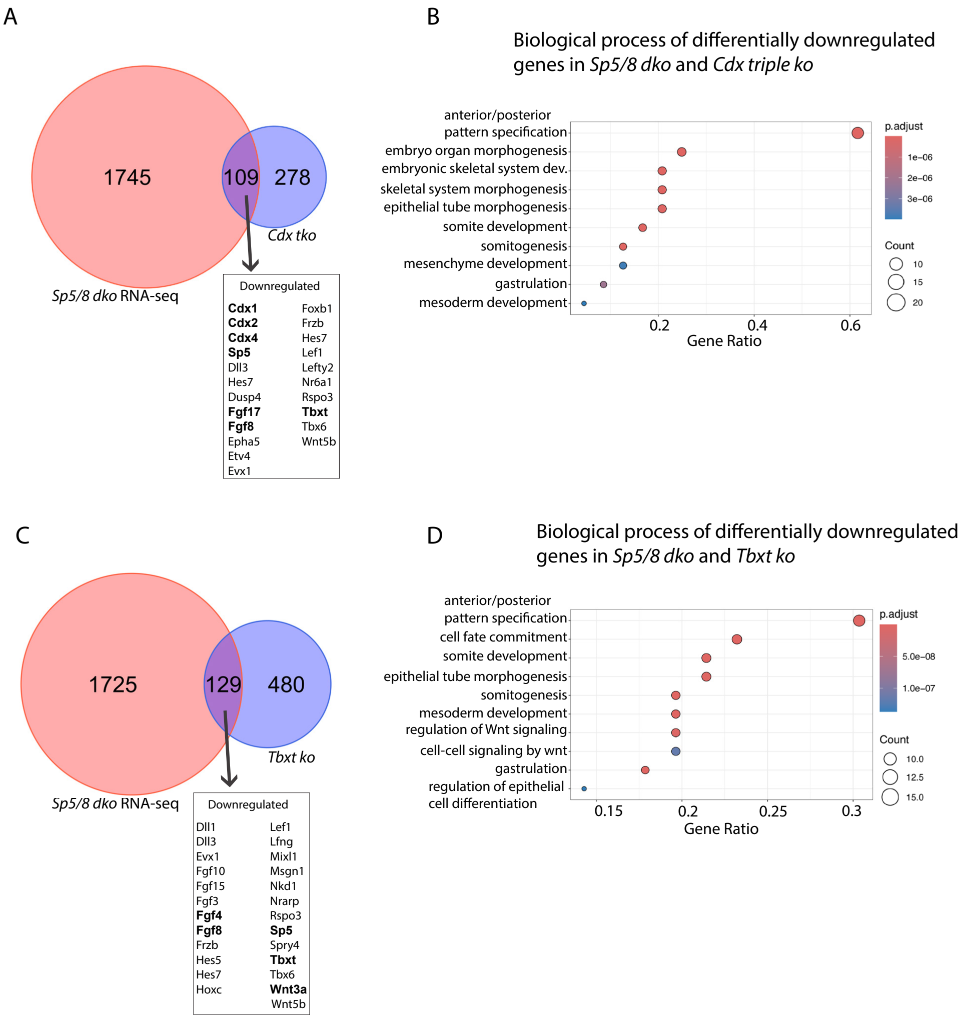

Supp. Fig.2. Comparisons of *Sp5/8dko* differentially expressed genes to Cdx-tko and Tbxt ko. Related to Fig.2 and 3.

A. Venn diagram showing the overlap of DEGs between the *Sp5/8* dko and *Cdx* tko (Amin et al., 2016). Select downregulated genes are featured, NMC-relevant genes are highlighted in bold.

B. GO biological processes terms of downregulated genes shared between *Sp5/8* dko and *Cdx* tko datasets.

C. Venn diagram showing the overlap of DEGs between *Sp5/8* dko and *Tbxt* ko (Koch et al., 2017). Key down regulated genes are highlighted.

bioRxiv preprint doi: https://doi.org/10.1101/2025.06.03.657492; this version posted June 4, 2025. The copyright holder for this preprint (which was not certified by peer review) is the author/funder. This article is a US Government work. It is not subject to copyright under 17 USC 105 and is also made available for use under a CC0 license.

D. GO biological processes terms of downregulated genes shared between *Sp5/8* dko and *Tbxt* ko datasets.

bioRxiv preprint doi: https://doi.org/10.1101/2025.06.03.657492; this version posted June 4, 2025. The copyright holder for this preprint (which was not certified by peer review) is the author/funder. This article is a US Government work. It is not subject to copyright under 17 USC 105 and is also made available for use under a CC0 license.

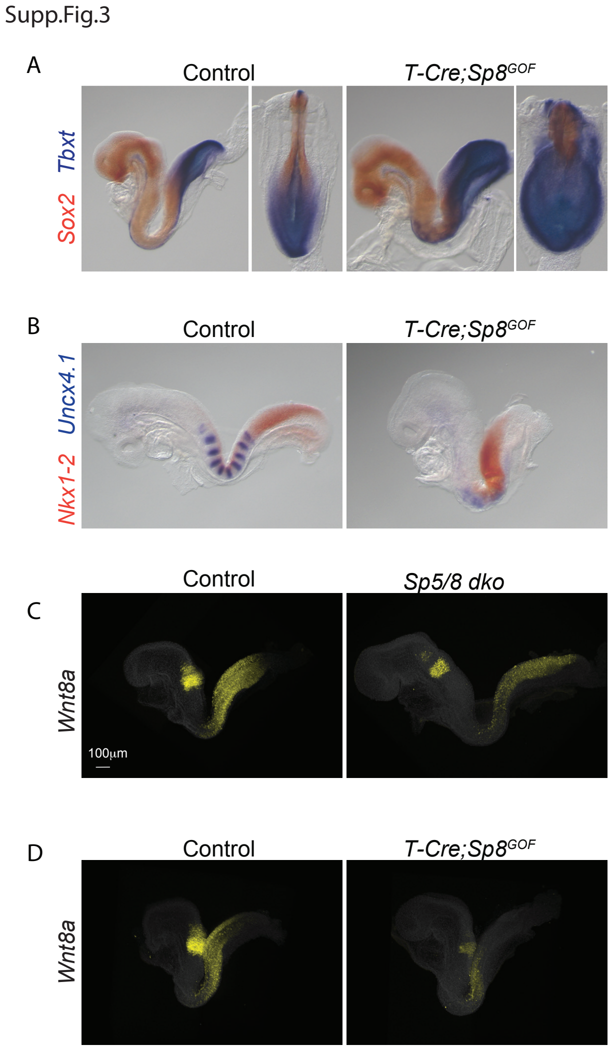

Supp. Fig.3. RNA in situ expression of NMC genes in *T-Cre-Sp8^{GOF}* and *Sp5/8* dko embryos. Related to Fig.4.

A-B. Two color whole mount in situ hybridization of E8.5 embryos for *Sox2* (orange) and *Tbxt* (purple) (A) and *Nkx1-2* (orange) and *Uncx4.1*(purple) (B) in control and *T-Cre; Sp8^{GOF}* embryos. Lateral and dorsal views are shown A and lateral view in B.

C-D. Lateral view of whole mount fluorescent in situ hybridization analysis for transcripts of *Wnt8a* (yellow) in *Sp5/8dko* embryos (C) and *T-Cre; Sp8^{GOF}* embryos at E8.5 (D).

bioRxiv preprint doi: https://doi.org/10.1101/2025.06.03.657492; this version posted June 4, 2025. The copyright holder for this preprint (which was not certified by peer review) is the author/funder. This article is a US Government work. It is not subject to copyright under 17 USC 105 and is also made available for use under a CC0 license.

Supp.Fig.4

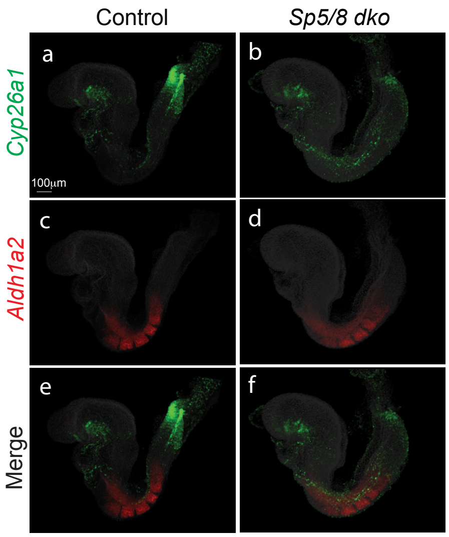

Supp. Fig.4. Whole mount fluorescent in situ hybridization analysis of RA pathway genes. Related to Fig.4.

Lateral view of embryos processed for *Cyp26a1* (green; a, b, e, f) and *Aldh1a2* (red, c, d, e, f) in E8.5 control and *Sp5/8dko* embryos.

bioRxiv preprint doi: https://doi.org/10.1101/2025.06.03.657492; this version posted June 4, 2025. The copyright holder for this preprint (which was not certified by peer review) is the author/funder. This article is a US Government work. It is not subject to copyright under 17 USC 105 and is also made available for use under a CC0 license.

Supp.Fig.5

A

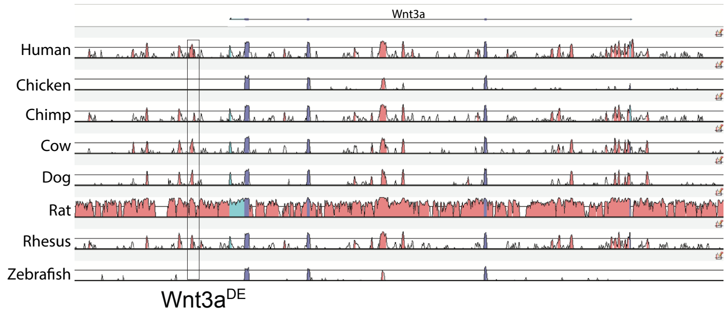

B

mm10, Chr11:59,243,723-59,244,188

CCCACTCAGACCCAAGCTCGCAGCTGTGGGCCTCAGAAGCAGCCTCTCTGGTGAGTTTA
CAGCCCCTCCCGCTGCCCTGGAGAAGGAGGAAATGAGCGGGAGGCCTGTTCACAGGTA
GTTGAGACAGCCCCCGAATAACCATATAAAAGGGCCGGAGGCTGGGGGCCCCACCCAGC
CAGGACAGGCTTGGCCATCCCCTTGCAAATCTCACACTCAGTAGCTTTGAAGTACTGGAA
AGGGTGTGGCCAACCCCCACCTTAGGGCCCCACTGACCCTCCCTTTTTCTGTCACCCCCTC
CCCTGGATGCCCACTTTCTGGGTCCTGGGGCTCAGTTTCCTCTCTTCAGAGACAGACTGGA
GGCTGTCACCATGGCCTGGCCTCCAGGAAAACCTACTCTTGGTCAGCTGGGGTGCAGCCA
GCTGTGGTTCCCACCCCTGGTTTGGGACTGAGAAAATGGACCTGAGGA

Sp5 motif   Cdx2 motif   Tcf/Lef motif   Tbx motif

Supp. Fig.5. Characterization of *Wnt3a^{DE}* using comparative genomics. Related to Fig.5.

A. Comparative genomics analysis of the mouse Wnt3a locus using Vista tools (Frazer et al., 2004). The conserved *Wnt3a^{DE}* is boxed.

B. Motif annotations from CIIIDER annotated against the 466 bp putative Wnt3a^{DE} sequence (Gearing et al., 2019). Wnt3a^{DE} is identified as -15696 intergenic peak (mm9; Chr11: 59057304 - 59057626 upstream of Arf1(see Supp. Data file-1).

bioRxiv preprint doi: https://doi.org/10.1101/2025.06.03.657492; this version posted June 4, 2025. The copyright holder for this preprint (which was not certified by peer review) is the author/funder. This article is a US Government work. It is not subject to copyright under 17 USC 105 and is also made available for use under a CC0 license.

Supp.Fig.6

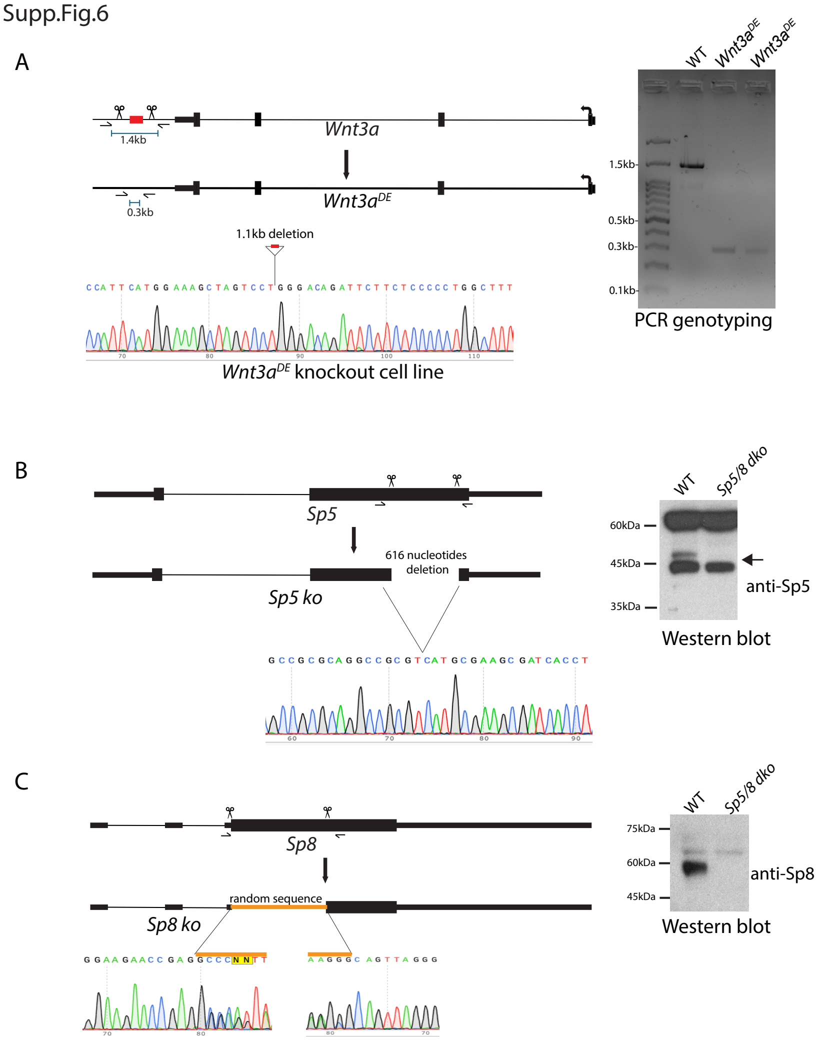

Supp. Fig.6. Generation of *Wnt3a^{DDE}* and *Sp5/8* dko ESCs using CRISPR/cas9 technology. Related to Fig. 6 and 7.

A. Schematic view of *Wnt3a^{DDE}* ko ES cell generation using CRISPR-Cas9 mediated deletion. Two guides (see Supp. data file-7) were designed to delete 1.1kb encompassing the 0.47kb *Wnt3a^{DE}*. Genotyping oligos were designed to amplify a 1.4kb fragment in wildtype and 0.3kb fragment in *Wnt3a^{DDE}* ko ESCs. PCR genotyping gel shows deletion of *Wnt3a^{DE}* in two different clones.

B-C. Generation of *Sp5/8dko* ES cells. B. Schematic of *Sp5 ko* using CRISPR-Cas9 mediated deletion in Exon-2 using two guides designed to delete 616 bases encoding the Zn-finger domains

bioRxiv preprint doi: https://doi.org/10.1101/2025.06.03.657492; this version posted June 4, 2025. The copyright holder for this preprint (which was not certified by peer review) is the author/funder. This article is a US Government work. It is not subject to copyright under 17 USC 105 and is also made available for use under a CC0 license.

of Sp5. ES cells were differentiated as EBs and treated with bFGF+CHIR for 24h. Sp5 protein was not detected in D3 cell extracts. C. Schematic view of *Sp8 ko* using CRISPR-Cas9 mediated deletion in Exon-3 using two guides designed to delete the coding region. Random sequence was inserted by NHEJ repair and these ES cells failed to express Sp8 protein in D3 EBs.

bioRxiv preprint doi: https://doi.org/10.1101/2025.06.03.657492; this version posted June 4, 2025. The copyright holder for this preprint (which was not certified by peer review) is the author/funder. This article is a US Government work. It is not subject to copyright under 17 USC 105 and is also made available for use under a CC0 license.

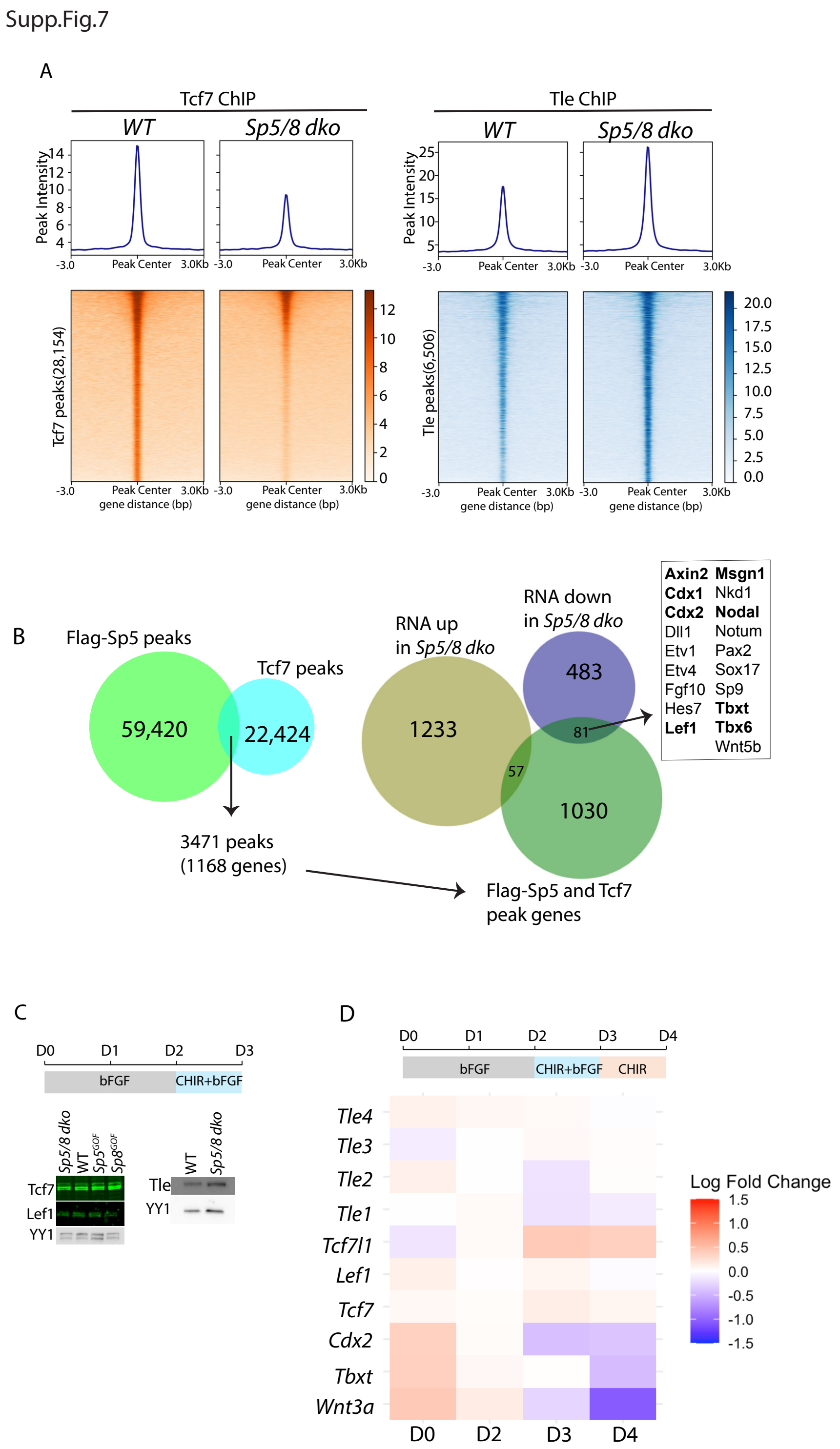

Supp. Fig.7. Comparative analyses of Flag-Sp5, Tcf7, and Tle ChIP-seq data sets. Related to Fig.7.

bioRxiv preprint doi: https://doi.org/10.1101/2025.06.03.657492; this version posted June 4, 2025. The copyright holder for this preprint (which was not certified by peer review) is the author/funder. This article is a US Government work. It is not subject to copyright under 17 USC 105 and is also made available for use under a CC0 license.

A. Metaplots and heat maps depicting 28,154 Tcf7 ChIP-seq peaks and 6,506 Tle peaks in WT and *Sp5/8dko* EBs.

B. (Left)Venn diagram illustrating the overlap between Flag-Sp5 and Tcf7 ChIP-seq peak sets. A total of 3,471 shared putative WREs, corresponding to 1168 unique genes, were identified. (Right) Venn diagram showing the intersection of the 1,168 genes associated with shared Flag-Sp5 and Tcf7 peaks and genes differentially expressed (up- or downregulated) in *Sp5/8dko* bulk-RNA-seq.

C. (Left) Western blot analysis depicting Tcf7 and Lef1 protein expression in NMCs differentiated in vitro from WT, *Sp5/8 dko*, $Sp5^{GOF}$ and $Sp8^{GOF}$ EBs. (Right) Tle protein expression in WT and *Sp5/8 dko* NMCs differentiated as EBs. YY1, loading control.

D. Quantitative PCR time course showing log fold change of RNA expression of *Tle1-4, Tcf7l1, Tcf7, Cdx2, Tbxt,* and *Wnt3a* in *Sp5/8 dko* versus WT EBs. RNA expression was normalized to *Gapdh* for all genes.

bioRxiv preprint doi: https://doi.org/10.1101/2025.06.03.657492; this version posted June 4, 2025. The copyright holder for this preprint (which was not certified by peer review) is the author/funder. This article is a US Government work. It is not subject to copyright under 17 USC 105 and is also made available for use under a CC0 license.

Supp.Fig.8

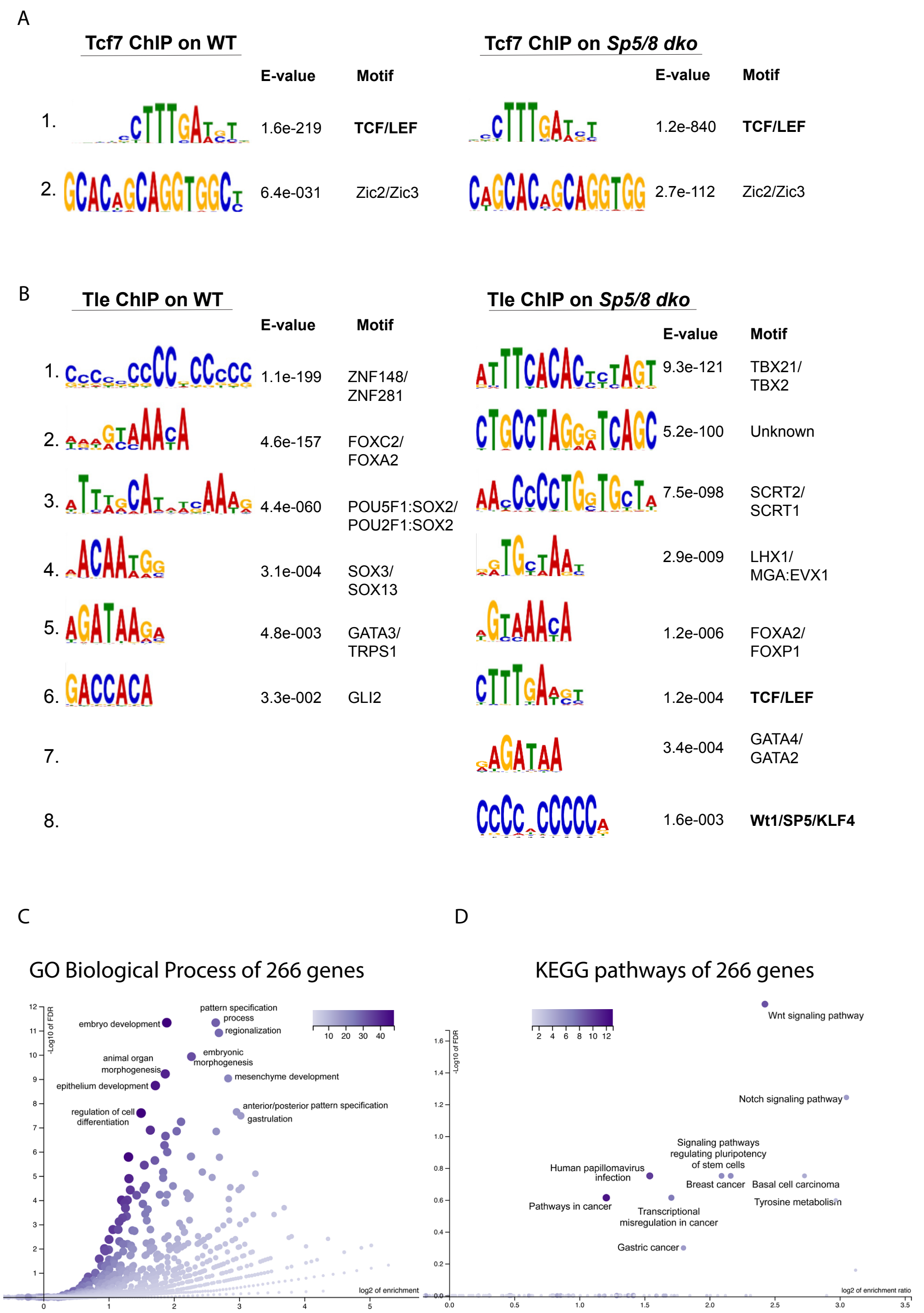

Supp. Fig.8. Motif and Over-representation analyses of F-Sp5, Tcf7, and Tle ChIPseq datasets. Related to Fig. 7.

bioRxiv preprint doi: https://doi.org/10.1101/2025.06.03.657492; this version posted June 4, 2025. The copyright holder for this preprint (which was not certified by peer review) is the author/funder. This article is a US Government work. It is not subject to copyright under 17 USC 105 and is also made available for use under a CC0 license.

A-B. Meme-ChIP motif analysis of peak sequences from Tcf7 (A) and Tle (B) ChIP-seq data in WT and *Sp5/8dko* cells. In Tcf7 ChIP-seq, the TCF/LEF motif was the most significantly enriched motif in both WT and *Sp5/8dko.* In Tle ChIP-seq, Znf motif and Tbx motif were most enriched in WT and *Sp5/8dko,* respectively, with additional enrichment of TCF/LEF and SP/KLF motifs in *Sp5/8dko* only.

C-D. Over-representation analysis (ORA) using WEB-based GEne SeT Analysis Toolkit on 266 overlapping genes corresponding to 286 F-Sp5 and Tcf7 overlapping WREs (as shown in Fig. 7B). Enrichment results are shown for (A) Gene Ontology (GO) Biological Process terms and (B) KEGG pathways.
